# The epigenetic regulation of centromeres and telomeres in plants and animals

**DOI:** 10.3897/CompCytogen.v14i2.51895

**Published:** 2020-07-07

**Authors:** Magdalena Achrem, Izabela Szućko, Anna Kalinka

**Affiliations:** 1 Institute of Biology, University of Szczecin, Szczecin, Poland University of Szczecin Szczecin Poland; 2 Molecular Biology and Biotechnology Center, University of Szczecin, Szczecin, Poland University of Szczecin Szczecin Poland

**Keywords:** cytosine methylation, histone code, non-coding RNA, pericentromeric, subtelomeric

## Abstract

The centromere is a chromosomal region where the kinetochore is formed, which is the attachment point of spindle fibers. Thus, it is responsible for the correct chromosome segregation during cell division. Telomeres protect chromosome ends against enzymatic degradation and fusions, and localize chromosomes in the cell nucleus. For this reason, centromeres and telomeres are parts of each linear chromosome that are necessary for their proper functioning. More and more research results show that the identity and functions of these chromosomal regions are epigenetically determined. Telomeres and centromeres are both usually described as highly condensed heterochromatin regions. However, the epigenetic nature of centromeres and telomeres is unique, as epigenetic modifications characteristic of both eu- and heterochromatin have been found in these areas. This specificity allows for the proper functioning of both regions, thereby affecting chromosome homeostasis. This review focuses on demonstrating the role of epigenetic mechanisms in the functioning of centromeres and telomeres in plants and animals.

## Introduction

The term epigenetics refers to a variety of processes that change gene expression independently of DNA sequence. An important feature of the epigenetic pattern is that it is stable and inherited through cell divisions, although it can be reversible (John and Rougeulle 2018). Epigenetics is crucial for the proper development, differentiation and functioning of cells. The epigenome may change under the influence of various environmental conditions and stimuli from inside the cell (Shi et al. 2017). This epigenome diversity is provided by numerous epigenetic mechanisms, including DNA methylation, post-translational histone modifications, chromatin remodeling, histone variants and ncRNA (non-coding RNA) interaction (Kabesch et al. 2010).

DNA methylation is of great importance among the epigenetic mechanisms that regulate gene expression in plants and animals. DNA methylation is associated with gene silencing (Kumar et al. 2018). Methylcytosine (5-mC) is the most common among the modified bases in the eukaryotic genome and is often referred to as the fifth DNA base. Methylation of cytosine in DNA involves the covalent attachment of a methyl group at position 5 of the cytosine pyrimidine ring (5-mC). Analysis of the DNA methylation profile of the human genome showed that mainly cytosines in CpG dinucleotides are modified. In plants, cytosine methylation in DNA occurs in the CHG sequential contexts (H = C, A, T) and asymmetrically in CHH (Zhang et al. 2008). Cytosine methylation in DNA is catalyzed by DNA methyltransferases. In mammalian cells, DNA methyltransferase (DNMT1) is responsible for maintaining the methylation pattern during replication, DNMT3A (DNA methyltransferase 3A) and DNMT3B (DNA methyltransferase 3B) for *de novo* methylation. In plants, MET1 (methyltransferase 1), DDM1 (decrease in DNA methylation 1), CMT1 (chromomethylase 1) and DRM2 (domain rearranged methyltransferase 2) DNA methyltransferases are necessary to maintain the correct methylation pattern (Ogrocká et al. 2014, Zhang et al. 2018).

Chromatin remodeling results from the action of ATP-dependent complexes that change the association of DNA with core histones and from modifications of histone proteins, affecting the availability of DNA (Kang et al. 2020). The remodeling complexes change the structure of chromatin by repositioning, evicting or restructuring the nucleosome. Some complexes are involved in the formation of condensed chromatin, others promote the binding of transcription factors to DNA. They are therefore involved in such important processes as DNA transcription and replication, DNA repair and DNA recombination (Clapier and Cairns 2009). Chromatin remodeling factors are involved in the development and differentiation of cells in plants and animals. Chromatin remodelers include several sub-families of ATP-dependent enzymes. Each of these subfamilies has a specific composition of domains and subunits that are involved in histone exchange, assembly and repositioning of nucleosomes (Kang et al. 2020).

Post-translational modifications of histone proteins are another important epigenetic mechanism. Histones (H2A, H2B, H3 and H4)_2_ are the basic protein component of the nucleosome that forms the core around which a DNA strand of about 146 bp is wrapped (Luger et al. 1997). The N- or C-terminal tails of histones undergo post-translational modifications. These modifications include arginine (R) methylation, methylation, acetylation, ubiquitination and sumoylation of lysine (K) as well as phosphorylation of serine (S) and threonine (T). The pattern of these modifications creates a histone code, which shows the transcription potential of this genomic region (Kabesch et al. 2010). Appropriate histone modifications are necessary for the proper course of such important cellular processes as: DNA repair, replication, mitosis, apoptosis and gametogenesis. Histones, through post-translational modifications, participate in the regulation of DNA packaging, affecting the availability of chromatin for transcription factors (Quina et al. 2006). Histone modifications can change the structure of chromatin by changing the physical properties of individual nucleosomes. This affects the interaction between the DNA molecule and histone and creates an open chromatin structure that is available for many protein factors, or a higher order chromatin structure that prevents these factors from binding. These modifications are strengthened by protein complexes that do not participate in chromatin modifications, but by influencing its remodeling, they are of great importance for the epigenetic gene regulation (Kim et al. 2012). An important role in regulating the structure of chromatin is also played by histone variants, which differ from canonical histones by the amino acid sequence. The presence of specific histone variants affects transcription regulation, chromosome segregation, DNA repair, cell cycle regulation and apoptosis (reviewed in Henikoff and Smith 2015).

Epigenetic regulators also include non-coding RNA (ncRNA). In epigenetic processes, the most important role among non-coding RNAs is played by those molecules that act in the RNAi (RNA interference) pathway and certain lncRNA (long non-coding RNAs, over 200 nt in length) (Kurokawa et al. 2009). Detailed studies of biogenesis and function of ncRNA have elucidated their activity at many levels, forming an integrated interacting network in the cell. They can regulate expression at both the gene and chromosome level (Amaral and Mattick 2008) and can act at transcriptional and post-transcriptional levels by interacting with promoters, enhancers or chromatin remodeling complexes (Kurokawa et al. 2009). However, their influence is not limited to the euchromatin, as exemplified by centromeric sequences, where ncRNAs are necessary for the assembly and proper functioning of both centromere and kinetochore (Bobkov et al. 2018).

Most of the presented epigenetic mechanisms are closely associated with each other to ensure stabilization and transmission of epigenetic patterns from cell to cell during cell divisions. They interact with each other in different ways. DNA methylation can promote changes in histone modification and vice versa. However, they can also change accidentally under the influence of stimuli coming from the internal and external environment (Kabesch et al. 2010). Epigenetic mechanisms do not act solely at the level of gene expression regulation. They also play a key role in maintaining genomic stability. They are involved in the regulation of centromeres, telomeres and silencing transposable elements (TE), which enables proper chromosome segregation, reduces excessive recombination between repetitive elements, and prevents TE transposition (Dupont et al. 2009).

However, there is a fairly close connection between epigenetic regulators and the spatial structure of the cell nucleus due to the fact that the organization of chromatin is epigenetically determined. In turn, the organization of chromatin influence the spatial structure of the cell nucleus. Based on the studies of the nucleus of mammalian cells, chromatin was divided into following compartments A – euchromatin, B – facultative heterochromatin (Solovei et al. 2016) and C – pericentromeric constitutive heterochromatin (Falk et al. 2019). It was shown that attractions between heterochromatic regions play crucial role in separation of the active from inactive parts of the genome in the nucleus. Constitutive heterochromatin, enriched with tandem repetitive sequences and transposable elements, located in the centromeric, pericentromeric or subtelomeric areas is the most enigmatic fraction of chromatin. Most of the heterochromatic regions remains unassembled due to their enrichment with the tandem repetitive sequences. The majority of the assembled mammalian genomes contain a 3 Mb Golden Path Gap (GPG) empty region around each centromere. However, gradually, more and more data on the composition of the sequence of constitutive heterochromatin regions are becoming available (Ostromyshenskii et al. 2018). Constitutive heterochromatin turns out to be surprisingly heterogeneous, characterized by plasticity, and its epigenetic regulators depend on the genomic context in which it is present. Although constitutive heterochromatin is gene-poor, its role turns out to be very significant (Saksouk et al. 2015).

The epigenetic nature of both centromeric and telomeric regions is not clearly defined. This is because these are regions built from repetitive sequences, which makes it difficult to accurately show epigenetic modifications of centromeres and telomeres. This review focuses on demonstrating how epigenetic mechanisms affect the functioning of centromeric and telomeric regions, taking into account differences in plants and animals.

## Centromere and pericentromere

The centromere was first described by Walther Flemming (1882), who observed that there was one region in the chromosome that was smaller in diameter than the remaining portion of the chromosome. Cytogenetic and molecular analyses demonstrated centromeres as heterochromatin chromosomal domains that control the formation of the kinetochore, a protein structure that interacts with the mitotic spindle, ensuring proper segregation of chromosomes (reviewed in Cleveland et al. 2003, Allshire and Karpen 2008, Salmon and Bloom 2017).

The simplest centromere with a length of 125 bp is found in *Saccharomyces cerevisiae* (Meyen, 1883). This simple, small centromere contains a single cenH3 (centromere specific histone 3) nucleosome, which binds a single microtubule during cell division, which is why this centromere type is called the “point centromere” (Pluta et al. 1995, Furuyama and Biggins 2007). Numerous studies have shown that not all eukaryotic organisms have monocentric chromosomes characterized by the presence of the primary constriction. In some species, microtubules of the mitotic spindle attach to the chromosome along its entire length (White 1973). Thus, two types of chromosomes are distinguished: monocentric chromosomes that connect to the microtubules of the spindle in a single region, and holocentric chromosomes, characterized by the presence of dispersed kinetochores that bind to spindle microtubules over their entire length (Wrench et al. 1994, Mandrioli and Manicardi 2012).

Holocentric chromosomes have been found in some plants (e.g. the genus *Luzula* Candolle and Lamarck, 1805) , animals (several arthopods and nematodes) and Rhizaria (Cavalier-Smith, 2002) (Allshire and Karpen 2008, Heckmann et al. 2013). It is believed that holocentromeres have been evolved from monocentromeres at least 13 times independently, and their organization varies among taxa (Melters et al. 2012). The type of DNA sequence responsible for the formation of dispersed centromeres is not yet fully elucidated. The sequences located in the holocentromeres are very diverse, including those that directly bind cenH3. In *Rhynchospora pubera* (Linnaeus, 1872) holocentromeres are enriched in specific satellite DNA sequences (Tyba) (which bind cenH3) and retrotransposons (Ribeiro et al. 2017). In *Caenorhabditis elegans* (Maupas, 1900) specific satDNA (satellite DNA) sequences that bind cenH3 are dispersed all over the genome (Subirana et al. 2018). In turn, no centromere-specific sequences were found in *Luzula elegans* (Lowe, 1838) (Heckmann et al. 2013). Hence, cenH3 probably binds not to specific sequences but to chromatin of appropriate status, indicating epigenetic regulation of holocentromers. The unusual structure of holokinetic chromosomes is also associated with the specific course of meiosis. Three types of meiosis can be distinguished in different species characterized by holocentric chromosomes: ‘chromosome remodeling’, ‘functional monocentricity’ and ‘inverted chromatid segregation’ (Heckmann et al. 2014, Lukhtanov et al. 2018). In *C. elegans* chromosome remodeling ensure chromosomes segregation typical for monocentric chromosomes. Other species have developed functional monocentricity, i.e. attachment of microtubules to one terminus of the chromosome, thus, holocentric chromosomes act as monocentric. These adaptations allow for a course of meiosis similar to canonical meiosis. In the first meiotic division, homologous chromosomes segregate, while sister chromatids are separated during the second meiotic division. However, many species with holokinetic chromosomes have developed an inverted meiosis, in which the order of major meiosis events is reversed, i.e. the sister chromatids are separated first (which results, among others, from the inability to maintain cohesion of sister chromatids up to AII (anaphase II) in holocentric chromosomes), followed by segregation of homologues (Heckmann et al. 2014, Lukhtanov et al. 2018).

In monocentric chromosomes of animals and plants the centromere region constitutes a segment from several kb to Mb in size, that contains satellite DNA with repeating monomers of ~100–400 bp (Melters et al. 2013). In general, chromosome centromeres in one species are characterized by the occurrence of a single family of sequence repeats (Zhong et al. 2002, Nagaki et al. 2003, Henikoff et al. 2015). This type of centromere restricted to a certain region is referred to as the regional centromere (Melters et al. 2013, Liu et al. 2015, Kursel and Malik 2016).

In plants, the centromeric region is composed of alternating tandem repeats and retrotransposons. For example, sequencing of maize centromeric DNA revealed two types of repetitive sequences in this region: satellite CentC (156 bp monomer) and retrotransposon CRM (centromeric *retrotransposon* of maize) sequences (Ananiev et al. 1998, Zhong et al. 2002, Birchler and Han 2009). In B chromosome of maize, an additional sequence was identified in this region known as B-repeat (Alfenito and Birchler 1993), flanked and interspersed with typical maize centromeric sequences, i.e. CentC and CRM (Jin et al. 2005, Lamb et al. 2005). A similar organization of sequences is found in rice centromeres, where the CentO satellite repetitive sequence (155 bp monomer) as well as the CRR (centromeric retrotransposon of rice) retrotransposon sequence are distinguished (Cheng et al. 2002); other examples are pBV repetitive sequences and r retrotransposon of the beetle family in *Beta vulgaris* (Linnaeus, 1753) (Zakrzewski et al. 2013). A combination of satellite repeats in association with retrotransposons in the centromere region was also detected in *Hordeum* (Linnaeus, 1753) (Houben et al. 2007), *Saccharum officinarum* (Linnaeus, 1753) (Nagaki and Murata 2005), *Brassica* (Linnaeus, 1753) (Wang et al. 2011), *Raphanus sativus* (Linnaeus, 1753) (He et al. 2015) and *Glycine* (Linnaeus, 1753) (Tek et al. 2010).

Human centromeres are characterized by the presence of satellite tandem repeats of ~171 bp in size, arranged “head-to-tail”, that are further arranged in higher order repeats (HOR). Individual monomers share 50–70% sequence identity, but HORs have 95–98% similarity (Warburton et al. 1996, Alcan et al. 2007). The functional core of the centromere is composed of highly homogeneous HORs, and, depending on the chromosome, spans a region from 0.5 to 5 Mb (Altemose et al. 2014), flanked by 500-kb segments, containing *L1* (*LINE1*, long interspersed nuclear *elements*) mobile elements (Schueler et al. 2001, Aldrup-MacDonald and Sullivan 2014). Within the human centromere, in the a satellite DNA sequences, 17-bp sequence motifs occur, referred to as the CENP-B box, which are recognized by centromere protein B (CENP-B) (Masumoto et al. 1993). This protein has an important role in maintaining stability and in the proper arrangement of centromere nucleosomes, because it binds with N-terminus of CENP-A (centromere protein A) and CENP-C (centromere protein C) (Fachinetti et al. 2015, Fujita et al. 2015). Human Y chromosome (Choo 2001) or neocentromeres (Fachinetti et al. 2015) are an exception, as the CENP-B box sequences and CENP-B proteins were not detected, while other centromeric proteins were present. It is known, however, that the lack of the CENP-B box in α-satellite sequences or mutations in these regions do not allow the formation of artificial chromosomes (Zhang et al. 2010). This suggests that CENP-B is not necessary for the centromere function, however, it contributes to its stabilization and maintenance (Schalch and Steiner 2017).

Centromeric DNA sequences are evolving relatively fast (Melters et al. 2013), which seems surprising considering the conservative function of the centromere (Henikoff et al. 2001, Rosin and Mellone 2017). Large differences in centromere sequences among wild *Oryza* species (Linnaeus, 1753) (Lee et al. 2005), cultivated *Canavalia* (Adanson, 1763) species (She et al. 2017), between related species of *Solanum tuberosum* (Linnaeus, 1753) and *S. verrucosum* (Schlechtendal, 1839) (Zhang et al. 2014), or within one species of *Pisum sativum* (Linnaeus, 1753) (Macas et al. 2007), can serve as examples. Hence, it is presumed that centromeres are not genetically determined by the occurrence of a specific DNA sequence, but they are rather epigenetically defined by characteristic modifications (Simon et al. 2015). The confirmation of this fact are neocentromeres, which act as centromeres at the new chromosomal site even if satellite sequences are not present there (Williams et al. 1998, Marshall et al. 2008). Although satellite DNA is an inherent element of centromeres, it is not required for the functioning of these regions (Willard 1990, Csink and Henikoff 1998). Nevertheless, repeated DNA is the preferred DNA environment for centromere formation, and if the neocentromere is formed in a region devoid of repetitive sequences, then they begin to gradually accumulate there (Han et al. 2009, Plohl et al. 2014).

The centromeric core, which provides the kinetochore attachment site, is flanked by pericentromeric regions. Pericentromeric chromatin stabilizes the centromeric core, inhibiting internal recombination between core repeat sequences (Hetrr and Allis 2005), and is responsible for the attachment of sister chromatids during cell division (Schalch and Steiner 2017), promoting bidirectionality and creating tension between them (Bernard et al. 2001, Sakuno et al. 2009, Yamagishi et al. 2010, Yi et al. 2018).

Pericentromeres, like the core centromere, mainly consist of repetitive sequences. Among the sequences included in pericentromeric DNA, there are satellite sequences, as well as transposons, LTR and non-LTR retrotransposons (Smurova and Wulf 2018). Typically, these regions are described as genetically inactive, although some of the sequences found in these regions, such as 5S rRNA genes are highly transcribed (Cloix et al. 2002, Simon et al. 2015). Pericentromeric sequences show both inter- and intraspecific variation (Charlesworth et al. 1994, Plohl et al. 2008).

## Epigenetic regulation of centromeres and pericentromeres

As previously mentioned, it is believed that satellite DNA is not essential for maintaining centromere structure and function. The term “centromere paradox” defines the fact that centromere sequences are very variable, while centromere function is conservatively maintained. However, as it turns out, centromere functionality does not result from the composition of the relevant DNA sequences, but the epigenetic mechanisms are responsible for it (Allshire and Karpen 2008). Epigenetic mechanisms play an important role in the establishment, maintenance and functioning of centromeres (Allshire and Karpen 2008) (Table 1). Centromere can be inactivated (Sullivan and Schwartz 1995, Han et al. 2006, Zhang et al. 2010), but also can switch from the inactive to active state, enabling transcription of ncRNA, which plays a role in the proper functioning of the centromere (Han et al. 2009). Centromeric ncRNAs interact with many proteins i.a. CENP-A (Rošić et al. 2014), CENP-B (Carone et al. 2009), CENP-C (Du et al. 2010), HJURP (Quénet and Dalal 2014) and AURORA B (Ferri et al. 2009). For example, centromere inactivation in dicentric chromosomes or activation of neocentromeres in non-centromeric regions were reported (Williams et al. 1998, Nasuda et al. 2005, Marshall et al. 2008, Topp et al. 2009).

**Table 1.** Epigenetic modifications of centromeric regions and their functions in plants and animals.

Epigenetic modification	Region	Function	Reference
histone variant cenH3CENP-A	centromeric	specifies centromere location essential for kinetochore assembly	Gieni et al. 2008
H3K4me1, H3K4me2, H3K36me2, H3K36me3	centromeric	maintenance of centromere stability RNA II pol activity recruitment of HJURP proteins CENP-A deposition	Yan et al. 2005 Foltz et al. 2009
H4K5ac and H4K12ac	centromeric	CENP-A deposition	Shang et al. 2016
H4K20ac	centromeric	required for transcriptional activity required for kinetochore formation in human and *Gallus* cells	Shang et al. 2016
H2AT133ph H2AT120ph	centromeric	recruitment of Shugoshin (Sgo1) protein prevents precocious separation of sister chromatids	Kawashima et al. 2010
monoubiquitinated H2B (H2Bub1)	centromeric	required for transcriptional activity provides structural integrity required for proper chromosome segregation	Sadeghi et al. 2014
H3K9me	pericentromeric	chromatin condensation ensures chromatid cohesion provides structural integrity	Gieni et al. 2008
H4K20me	pericentromeric	chromatin condensation provides structural integrity	Gieni et al. 2008 Hori et al. 2014
H3K27me	pericentromeric	transcriptional repression of transposable elements	Jacob et al. 2010 Feng et al. 2017
H3 and H4 lysine residues acetylation	pericentromeric and centromeric	increase in chromatin compaction heterochromatin integrity	Gieni et al. 2008
Cytosine methylation of DNA	pericentromeric and centromeric	chromatin condensation provides structural integrity	Gieni et al. 2008 Song et al. 2013

The results of studies on the epigenetic regulation of centromeric regions are ambiguous. The difficulty in studying these regions is caused by the fact that centromeres in most multicellular eukaryotes are formed of numerous copies of repetitive sequences (Henikoff et al. 2001). Identification of individual epigenetic modifications is particularly difficult if the sequences of the same family of repeats have different epigenetic markers. For this reason, many studies do not present unequivocal results. There is also a limitation in the selection of methods to study these regions. For example, standard methods used to map DNA methylation, including high-throughput techniques based on microarrays and WGBS sequencing (bisulfite sequencing-based platforms), do not allow to assess methylation within highly repetitive DNA sequences. Therefore, in this case, immunofluorescence (IF) analysis is often used in combination with FISH (fluorescence *in situ* hybridization) on stretched DNA fibers (Koo et al. 2011).

Many studies on centromere chromatin in *Arabidopsis thaliana* (Linnaeus, 1753) have shown that it forms chromocentres in the interphase nuclei, it is rich in H3K9me2, characterized by DNA hypermethylation and enrichment in histone variant H2A.W (Probst et al. 2003, Stroud et al. 2013, Yelagandula et al. 2014). However, comprehensive IF studies using anti-5-methylcytosine antibody showed that the DNA in centromeric region is unmethylated. IF on the stretched fibers of the early pachytene chromosomes confirmed these observations, indicating that DNA sequences (178 bp tandem repeats) in the core regions with cenH3 were differently methylated than in the flanking pericentric regions. Regions in which cenH3 is present, and directly adjacent regions, are unmethylated or significantly less methylated, while the remaining 178 bp repeats are highly methylated. Thus, DNA sequences in centromeric chromatin are hypomethylated compared to the sequences found in the flanking pericentric chromatin (Zhang et al. 2008). In addition, a correlation was found in Arabidopsis between the occurrence of 5mC and H3K9me2 in centromeric regions. Similar results were obtained while studying centromeric regions in maize. The methylation status of centromeric CentC repeats in maize is variable, whereby, similarly to Arabidopsis, DNA sequences associated with cenH3 in maize are hypomethylated (Koo et al. 2011).

In contrast, studies on centromeres in rice have shown that DNA sequences in a functional centromere can be both hypo- and hypermethylated. DNA methylation patterns appear to be correlated with specific sequence motifs (CG, CHG, CHH) in centromeric DNA (Yan et al. 2010). Detailed studies of the centromeric maize region have shown that there is a tendency of increased DNA methylation in CG and CHG motifs towards the centromere and decreased towards the chromosomal arms. This was also observed in *Populus trichocarpa* (Torrey et Grey, 1851) (Feng et al. 2010, Zemach et al. 2010). In turn, CHH methylation was relatively similar in different maize chromosomal domains, which was also confirmed by studies concerning rice centromere (Feng et al. 2010). Although general methylation level was similar in centromeres and pericentromeres, a slight increase in CG methylation and a decrease in CHG was observed in the centromeric core, with a marked difference between centromeres (Gent and Dawe 2012). This variation may result from the relative differences in the size of CentC sequence stretches in the individual centromeres (Jin et al. 2005).

Research on the level of DNA methylation in medaka fish (*Oryzias latipes* Temminck et Schlegel, 1846) demonstrated that centromeres are mainly hypermethylated, but have hypomethylated subregions (Ichikawa et al. 2017). It was found that DNA methylation patterns in centromeres were not correlated with the phylogenesis of centromeric sequences, but the hypo-/hypermethylated regions in individual chromosomes evolved independently by acquiring a unique sequence composition. In turn, examining methylation level in mouse cells, it was found that it depended on the type of tissue being tested. The highest level was observed in somatic cells, intermediate in sperm and the lowest in egg cells (Yamagata et al. 2007).

**Figure 1.**
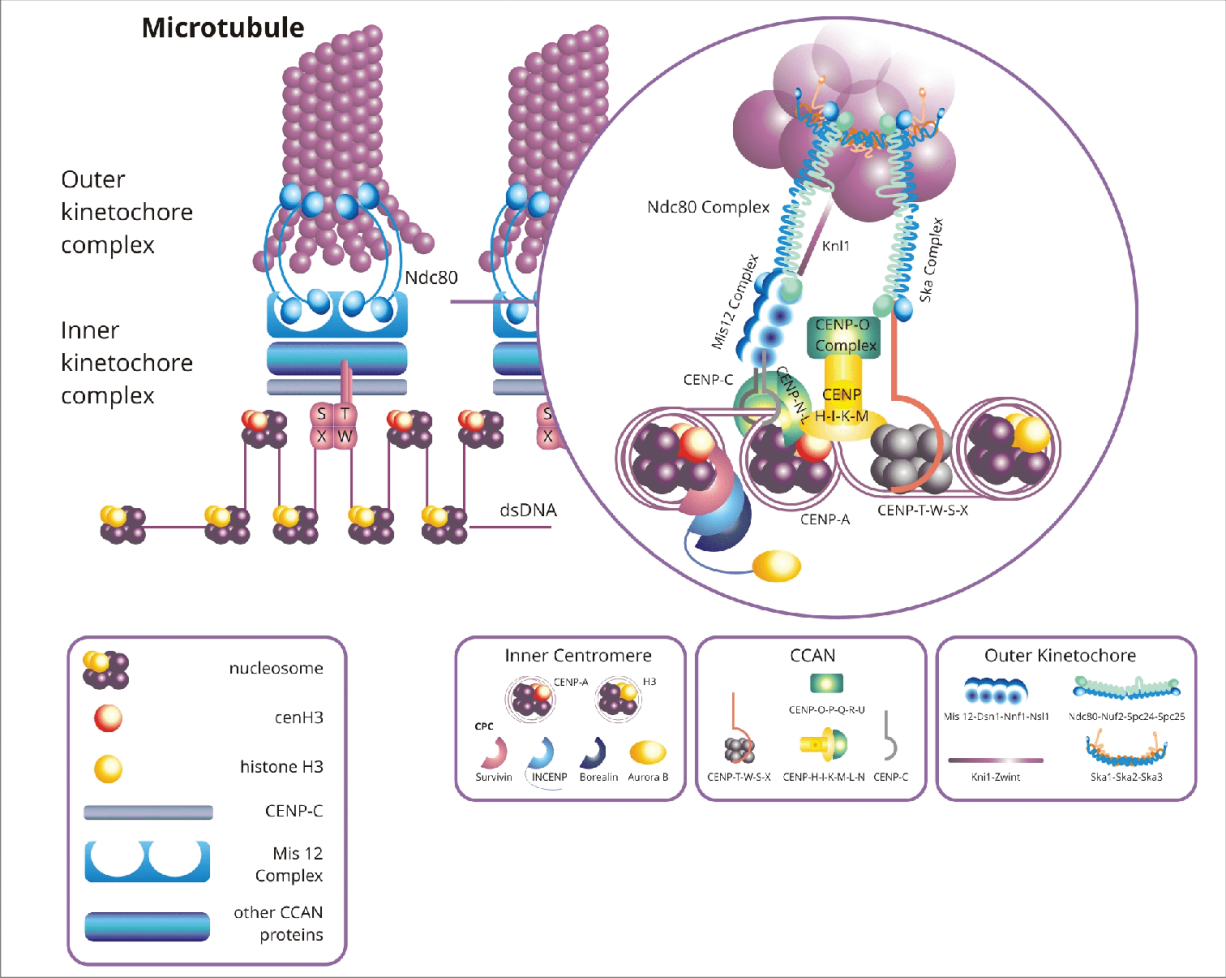
Model of the vertebrate mitotic centromere/kinetochore complex. Kinetochores assemble on chromatin marked by CENP-A containing nucleosomes. CENP-A nucleosome binds chromosomal passenger *complex (CPC), which consists of four proteins*: kinase Aurora B, INCENP, Survivin and Borealin. The kinetochore is composed mainly of CCAN (constitutive centromere-associated network) and Knl1-Mis12-Ndc80 complexes. The presence of CENP-A allows the recruitment of CCAN, which is a complex consisting of 16 centromeric proteins: CENP-C, CENP-T-W-S-X, CENP-H-I-K-M, CENP-N-L and CENP-O-P-Q-R-U. CENP-C and CENP-N bind CENP-A. The CENP-T-W-S-X complex creates a unique nucleosome-like structure that allows DNA binding in centromeric chromatin. CENP-N-L and CENP-H-I-K-M have regulatory roles. CENP-H-I-K-M-L-N help recruit CENP-C. CENP-C binds to the Mis12 complex, which then recruits Knl1 proteins interacting with microtubules and the Ndc80 complex. Ndc80 – kinetochore complex component (the complex consists of Ndc80-Nuf2-Spc24-Spc25 proteins); cenH3 – centromere specific histone 3 or histone H3 variant found at the centromere, CENP-A – centromere protein A, centromere specific histone 3 or histone H3 variant found at the centromere; CENP-C – centromere protein C; Mis 12 Complex – complex of the core kinetochore (the complex consists of *Mis12*-*Dsn1*-*Nnf1*-*Nsl1 proteins)*; Knl1 – *kinetochore* scaffold 1; Zwint – kinetochore proteins; CCAN – constitutive centromere-associated network, CPC – chromosomal passenger complex (consisting of Borealin, Survivin, INCENP, and the Aurora B kinase), INCENP – Inner Centromere Protein; Ska Complex – spindle and kinetochore associated (the complex consists of Ska1-Ska2-Ska3 proteins).

Centromeric chromatin (CEN) is characterized by the presence of specific histone H3 variant – cenH3 (CENP-A in mammals, CID (*centromere identifier*) in *Drosophila melanogaster* (Fallén, 1823), cenH3 in plants) (Steiner and Henikoff 2015). In multicellular eukaryotes, centromeres consist of alternating blocks of nucleosomes containing H3 or cenH3 (Blower et al. 2002, Sullivan and Karpen 2004, Alonso et al. 2007). The cenH3 nucleosomes recruit complexes that directly bind to cenH3, which in turn allows the attachment of numerous centromeric proteins termed CCAN (constitutive centromere-associated network) (Foltz et al. 2006, Carroll et al. 2009) (Fig. 1). The HJURP chaperone protein (Holliday junction recognition protein) is involved in the process of CENP-A deposition and complex formation between CENP-A and H4 (Shuaib et al. 2010). The structure of human CENP-A differs from canonical H3 histone, *inter alia*, by loop 1, which contains two additional amino acid residues (Arg80 and Gly81), affecting centromere chromatin stabilization (Tachiwana et al. 2011, González-Barrios et al. 2012). CENP-A shows only 50% homology to H3 amino acid sequence. There is also variation in length and sequence of N- and C-termini among these proteins (Malik and Henikoff 2003), simultaneously the C-terminus retains the hydrophobic region necessary for interaction with CENP-C (Kato et al. 2013). Moreover, it was shown that around the nucleosome containing CENP-A only 121 bp of the DNA is wrapped, 13 bp from both DNA ends are invisible in the crystal structure suggesting highly flexible ends (Tachiwana et al. 2011, Roulland et al. 2016). This structure disrupts the binding of histones H1 with the nucleosomes, allowing a more open configuration of the chromatin, which in turn enables the attachment of the CCAN complex (Roulland et al. 2016). Studies have shown that there are structural differences between CENP-A/H4 and H3/H4 heterotetramers (reviewed in Verdaasdonk and Bloom 2011). The presence of the CENP-A protein in the nucleosome ensures its more compact and rigid structure (Black et al. 2007). Similarly to CENP-A, plant centromeric cenH3 is characterized by significant variability between species (Malik and Henikoff 2009). cenH3 has a conserved histone-fold domain (HFD), instead the most significant differences in the structure of this protein in relation to H3 occur at the N-terminus (Ravi et al. 2010; Lermontova et al. 2014). This may be due to the fact that the C-terminus of cenH3 is responsible for histone H4 binding, which allows the formation of stable nucleosomes (Feng et al. 2019).

In human CEN chromatin, nucleosomes containing the CENP-A variant alternate with nucleosomes with the canonical histone H3. Histones H3 in this region undergo methylation at lysine positions 4 and 36 (H3K4me1, H3K4me2, H3K36me2, H3K36me3), characteristic of transcriptionally active chromatin. They affect RNA pol II (RNA polymerase II) activity and play an important role in the recruitment of HJURP proteins that participate in the CENP-A deposition (Bergmann et al. 2011, Duda et al. 2017). The absence of H3K4me2 in the centromere of artificial human chromosomes resulted in the inactivation of this centromere (Bergmann et al. 2011), which shows a functional link between epigenetic modification of CEN chromatin and maintaining centromere stability. Similarly, in plants, dimethylation of histone H3 at lysine 4 (H3K4me2) is a common modification in the centromeric H3 subdomains (Wu et al. 2011), which was not observed, for example, in the cenH3 subdomains of rice. It has even been hypothesized that the transcribed sequences located in the rice centromere can be a barrier preventing the introduction of cenH3 into the region of H3 subdomains. This separation of the cenH3 and H3 subdomains in the centromere core may be necessary for the formation of three-dimensional structure and functioning of rice centromere (Wu et al. 2011).

Interestingly, CEN is not usually associated with the presence of H3K9me2 or H3K4me3 heterochromatin markers, although H3K9me3 modification has been shown in this region to be associated with transcription repression (Bergmann et al. 2012). This illustrates that CEN chromatin can be both silenced heterochromatin as well as active euchromatin (Sullivan and Karpen 2004), however, it is important that the balance between them is preserved. Introduction of repressors or activators of transcription in artificial chromosomes disrupts the balance between modifications such as H3K4me2 and H3K9me3, which leads to the loss of the centromere function (Nakano et al. 2008).

In maize centromeres, the presence of histone post-translational modifications associated with transcriptional activity, such as histone H4 acetylation and H3K4me2, has been revealed. It was indicated that centromeres in this species are organized as euchromatin regions flanked by pericentromeric H3K9me2-enriched heterochromatin (Yan et al. 2005). Histone H4 acetylation (H4K5ac and H4K12ac) was also detected in *Gallus* (Brisson, 1760) cells as a modification necessary for CENP-A deposition (Shang et al. 2016). It was shown that H4K20ac is essential for transcription of ncRNA, which is necessary for the deposition of CENP-A and kinetochores assembly in human and *Gallus* cells (Sullivan and Karpen 2004, Wang et al. 2008, Bergmann et al. 2011, Hori et al. 2014). Moreover, for the transcription of centromeric DNA monoubiquitination of lysine 119 in histone H2B (H2BK119ub1) must occur (Zhu et al. 2011, Sadeghi et al. 2014). It is mediated by the ubiquitin ligase E3 RNF20 (ring finger protein 20) in humans or Brl1 in *Schizosaccharomyces pombe* (Lindner, 1893) (Sadeghi et al. 2014). The H2BK119ub1 modification interacts with many proteins such as RNA pol II and SWI/SNF (switch/sucrose non-fermentable) protein complexes (Shema-Yaacoby et al. 2013), which contributes to the formation and maintenance of transcriptionally active chromatin. This modification also affects centromere integrity and accurate chromosome segregation. It has been shown that the decrease in RNF20 level results in H2BK119ub1 deficiency in this region, which in turn causes heterochromatin formation, thereby reducing the transcription of the centromeric DNA sequence and resulting in an abnormal chromosome segregation in human and *S. pombe* (Lindner, 1893) cells (Sadeghi et al. 2014, Zhang et al. 2017).

**Figure 2.**
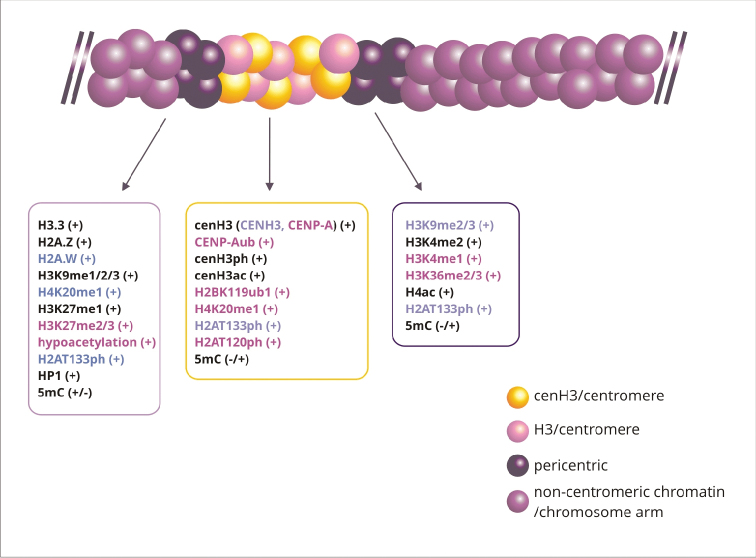
Epigenetic modifications in centromeric and pericentric chromatin. Centromeres consist of alternating blocks of nucleosomes containing H3 or cenH3. At pericentric sites, only H3-containing nucleosomes are present. Epigenetic markers in centomere and pericentromere regions characteristic for both plants and animals are marked with black color, only for plants with violet color, only for animals with rose color. (+) epigenetic marker always present; (-/+) epigenetic modification present or absent.

CENP-A is less likely to undergo post-translational modification than canonical histone H3 (Fig. 2). This is due to, *inter alia*, the lower lysine content in CENP-A. In histone H3, up to 17 different types of post-translational modifications were found (Xu et al. 2014), whereas only four modifications were detected in CENP-A: methylation, acetylation, phosphorylation and ubiquitination (Srivastava and Foltz 2018). The most characteristic CENP-A modifications are Gly1 trimethylation, Ser 7, 16, 18 and 68 phosphorylation and monomethylation, acetylation and *ubiquitination* of lysine 124. These CENP-A-specific modifications, play an important role in chromosome segregation during cell division, because they regulate CENP-A deposition in centromeric chromatin and participate in CCAN recruitment (Srivastava and Foltz 2018).

It has long been believed that centromeric chromatin is transcriptionally inactive because it is formed mainly by satellite sequences. It is now known that CEN transcription is mediated by RNA pol II, which was detected in centromeric regions in both *S. pombe*, *Drosophila*, mouse, human, *Zea* (Linnaeus, 1753), *Oryza* (Linnaeus, 1753) and neocentromeres, as well as in CEN of human artificial chromosomes (HAC) (Chueh et al. 2009, Ferri et al. 2009, Ohkuni and Kitagawa 2011, Chan and Wong 2012, Podgornaya et al. 2013, Quénet and Dalal 2014, Rošić et al. 2014). The important role of transcription in centromere integrity was shown by numerous studies on its inhibition, which resulted in the loss of centromere function (Quénet and Dalal 2014, Rošić et al. 2014, Sadeghi et al. 2014). Many genes have been identified in the centromeric regions of various plants, including rice (Jiang 2013) and *A. thaliana* (May et al. 2005). Transcribed centromeric elements can activate the process of RNAi by forming siRNA (small interfering RNA) and affecting both DNA and histone modifications in the centromeric region (Lippman and Martienssen 2004). Studies also showed transcriptional activity of centromeric retrotransposons that affect the formation, stabilization and functioning of centromeres (Jiang et al. 2003, Topp et al. 2004). An example is the CRM transcript in maize, which contributes to the stabilization of centromere chromatin (Topp et al. 2004) or the CRR transcript in rice that is involved in the formation and maintenance of centromeres through RNAi pathway (Neumann et al. 2007). The additional evidence, that transcription of centromeric DNA is common, is the presence of H3K4me2 modification in this region of many plants (onion, rice, Arabidopsis, maize). Maintaining CEN chromatin in the active state and its transcription is also necessary for the replacement of histone H3 with cenH3 (Quénet and Dalal 2014, Bobkov et al. 2018). The lack of centromeric transcripts leads to disturbances during mitosis (Quénet and Dalal 2014). Centromeric chromatin is transcriptionally active even during mitotic division (Chan et al. 2012), which ensures stability of kinetochores and coherence of centromeres (Liu et al. 2015). Phosphorylation of centromeric histone H2A (H2AT120ph in animals, H2AT133ph in plants) by the Bub1 (budding uninhibited by benzimidazoles 1) kinase is required for the recruitment of the Shugoshin protein (Sgo1). This protein ensures chromatid coherence in internal centromeres (Kawashima et al. 2010). Sgo1 interacts with RNA pol II and is directed to the inner centromere between two sister chromatids. The open chromatin structure in the centromeric region allows binding of the Sgo1 protein to cohesin and provides protection against premature chromatid separation (Kang et al. 2011, Liu et al. 2015). Initiation of centromeric DNA transcription must be preceded by chromatin remodeling. An important factor in this process is a histone chaperone, FACT (facilitates chromatin transcription) (reviewed in Reinberg and Sims 2006). FACT allows transcription through the destabilization of nucleosomes, allowing polymerase to access DNA (Belotserkovskaya et al. 2003). After polymerase passes, it allows a return to the earlier chromatin structure (Jamai et al. 2009).

It has also been proven that the region directly adjacent to the centromere plays a role in sister chromatid cohesion (Bernard et al. 2001, Steiner and Henikoff 2015). Between the prophase and anaphase, sister chromatids are kept together in pericentromeres after cohesins are removed from other chromosome regions (Nasmyth and Haering 2009). There are known various epigenetic mechanisms associated with chromatin silencing that provide cohesion maintenance in pericentromeres (HP1-*heterochromatin protein 1*, H3K9me3, RNAi) (Mosch et al. 2011). Changes in this region may lead to impairment of proper chromosome segregation (Allshire et al. 1995, Steiner and Henikoff 2015). However, there are hypotheses that this heterochromatin region is necessary to establish the centromere, but is not required to retain it (Folco et al. 2008). In addition, studies on neocentromeres, which can form in euchromatin areas, indicate that pericentromeric heterochromatin (PHC) is not necessary for the proper functioning of the centromere (Shang et al. 2013). Nevertheless, it is believed, that pericentromeric heterochromatin regions may play a role in preventing the centromere from spreading to adjacent regions (Sullivan 2002). From an epigenetic point of view, pericentromeres show a greater similarity to centromeres than to other chromosomal regions. This is reflected in siRNA transcription, DNA methylation and some post-translational modifications of histones. Although there is evidence that centromeres may function independently of pericentromeres, as found, for example, in studies conducted on *S. cerevisiae* (Weber et al. 2004), there is a strong interdependence of these two regions (Han et al. 2006).

Histones in pericentric chromatin are mostly hypoacetylated, which causes chromatin condensation. Pericentromeric areas are characterized by the presence of histone variants H3.3 and H2A.Z (Drané et al. 2010, Santenard et al. 2010), modifications of histones such as mono-, di- and trimethylation of H3K9, H3K27 and H4K20 (Feng et al. 2017) and a high level of 5-mC in DNA (Song et al. 2013). These modifications are characteristic of transcriptionally inactive chromatin and play a role in the silencing of genetic mobile elements occurring abundantly in these chromosomal regions (Roudier et al. 2011, Rose and Klose 2014, Feng et al. 2017). For example, monomethylation of lysine 27 in histone H3 is associated with constitutive repression of transcription. This was confirmed by the study of pericentromeric regions of polytene chromosomes of *Drosophila*. They correspond to green – inactive (the division of chromatin into the following shades: red, yellow, blue, green and black; according to Filon et al. 2010) or ruby chromatin (the division of chromatin into the following shades: aquamarin, lazurite, malachit and ruby; according to Zhimulev et al. 2014), which is characterized by H3K27 methylation as well as SU(VAR)3-9 and HP1 presence (Boldyreva et al. 2017). Loss of H3K27 methylation in the pericentromeric regions causes transposons reactivation (Jacob et al. 2010). This may result in a cancer or other diseases such as ICF (immunodeficiency, centromere instability, facial anomalies). ICF is a rare autosomal recessive disease characterized by a lack of DNMT3B activity. DNA methylation depletion results in the loss of repressive histone modifications (often H3K27me3) and the appearance of modifications characteristic of euchromatin (H3K9ac, H3K4me), which further leads to reactivation of transposons (Jin et al. 2008).

A characteristic protein of this region is HP1 or its homologs (Guenatri et al. 2004, Cam et al. 2005), which affect the stabilization and maintenance of the heterochromatic state (Saksouk et al. 2015) of pericentromeric regions. The HP1 protein interacts with the Suv39h histone methylation kinase, which catalyzes the trimethylation of lysine 9 in H3 (Aagaard et al. 1999, Grewal and Jia 2007). In mice, it has been found that Suv39h deficiency results in a lack of H3K9me3, disrupting the occurrence of HP1 in the pericentromeric heterochromatin, which in turn translates into abnormal chromosomal segregation (Peters et al. 2001, Maison et al. 2002). The heterochromatic nature of the pericentromeric region is also confirmed by the analysis of marker gene expression. Inserted into the pericentromeric region, they are transcriptionally silenced, while the insertion of the same genes into the CEN region shows a significantly weaker silencing effect (Allshire et al. 1995).

The analysis of human neocentromeres that showed centromere functioning without satellite repeats (although they had a slightly higher AT content, from 59.9 to 66.1% compared to genomic average of 59%). The acquisition of centromeric function by a chromatin region without changing the DNA sequence was called the “centromerization” phenomenon (Choo 2000). Such neocentromeres, formed outside the centromeric regions, while maintaining the characteristics of the original centromere without the underlying centromere DNA, were also observed in animals and plants (*Gallus* (Brisson, 1760), *Equus* (Linnaeus, 1758), *Solanum* (Linnaeus, 1753), *Hordeum* (Linnaeus, 1753), *Avena* (Linnaeus, 1753) and *Zea* (Linnaeus, 1753)) (Nasuda et al. 2005, Ishii et al. 2008, Kagansky et al. 2009, Topp et al. 2009, Piras et al. 2010, Gong et al. 2012, Fu et al. 2013, Shang et al. 2013). The existence of neocentromeres and rapid evolution of centromeric DNA suggest that these are epigenetic mechanisms, rather than DNA sequence itself, that determine centromere functions (Piras et al. 2010).

Studies on dicentric chromosomes also support this fact. Dicentric chromosomes are the result of genomic rearrangements placing two active centromeres on the same chromosome. Most dicentric chromosomes are unstable and only due to epigenetic mechanisms, which deactivate one of the centromeres, monocentric chromosomes can be formed that normally segregate during cell division (Sullivan and Schwartz 1995, Chiatante et al. 2017). If one of the centromeres is not turned off, the chromosome breaks during division. DNA sequences of the active and inactive centromeres of dicentric chromosomes are almost identical, but the centromere activity states are completely different. Centromere inactivation on the dicentric chromosome is carried out by H3K27me2 and H3K27me3. Smaller centromeres appear to be inactivated more frequently than the larger ones (Han et al. 2009). It was confirmed by analyses of dicentric chromosomes in plants e.g. *Zea mays* (Linnaeus, 1753), (Han et al. 2006), *Oryza sativa* (Linnaeus, 1753) (Wang et al. 2013) and in humans. This explains some processes regarding the formation and maintenance of neocentromeres in human, because neocentromeres are always smaller than the native ones. If small centromeres are more susceptible to inactivation compared to larger ones, then most of the newly formed neocentromeres will be inactivated during subsequent cell divisions (Zhang et al. 2010).

Evolutionary repositioning or shift of the centromere along the chromosome with its function, leading to the formation of new evolutionary centromeres (ENCs), is another phenomenon that shows the epigenetic nature of these structures. This phenomenon was observed in primate chromosomes, other placental, marsupials and birds (Montefalcone et al. 1999, Ventura et al. 2007, Piras et al. 2010, Zlotina et al. 2012). The beginning of repositioning causes the loss of the function of the original centromere, followed by epigenetic changes in the non-centromeric position, leading to the formation of a new functional centromere in the chromosome region devoid of satellite DNA (Montefalcone et al. 1999). The resulting neocentromere may gradually accumulate repetitive DNA sequences through recombination mechanisms during evolution (Piras et al. 2010). Accumulation of these sequences probably ensures the stabilization of the centromere during cell division (Marshall et al. 2008), facilitates incorporation of histone cenH3 (Steiner and Henikoff 2015) and the accuracy of chromosomal segregation (Piras et al. 2010). All these reports shed more light on the role of satellite sequences. Despite their heterogeneity between species, a common pattern of structural DNA motifs required for centromere specification begins to be noticed (Black and Giunta 2018, Oliveira and Torres 2018). This hypothesis is supported by the fact that *de novo* chromosome formation revealed preferential centromere occurrence in areas built of tandem repeats (Grimes et al. 2002, Masumoto et al. 2004, Nagaki et al. 2004, Han et al. 2009).

## Telomere and subtelomere

Telomeres are specialized structures located at the ends of linear eukaryotic chromosomes. Their function is to protect the ends of chromosomes from inappropriate enzymatic degradation. They are also responsible for chromosome localization in the cell nucleus and transcription regulation of genes located near telomeres (Deng et al. 2008, Fojtová and Fajkus 2014). Telomeres also protect chromosomes from fusions, formation of dicentric chromosomes and homologous recombination (Artandi and DePinho 2010). While telomere function has been well known for a long time, the role of the subtelomeric region is still being investigated. It is indicated that subtelomeres support telomeres in their function, because they may affect processes such as cell cycle regulation, cell aging, motility and chromosomal localization in the nucleus (Riethman et al. 2005).

Due to the important functions they perform in the cell, telomeres are evolutionarily conserved regions and their structure is only slightly different in individual species. However, the length of telomeric sequences shows individual, tissue and cellular variability (Marión and Blasco 2010). Telomeres contain a double-stranded region composed of tandem DNA repeats, which can be described by the following formula: 5’-T_x_(A)G_y_-3’ (x, y – number of repeats) and single-stranded free 3’ end rich in guanine (G-overhang) (Wang and Zakian 1990, Smogorzewska and de Lange 2004), whose length varies from 16 to 200 nt depending on the species (Kazda et al. 2012). There are, however, exceptions from the above formula for telomere monomers, e.g. in *Allium cepa* (Linnaeus, 1758) this is the (CTCGGTTATGGG)_n_ sequence (Fajkus et al. 2016), in *Genlisea* (Bentham and Hooker, 1883) two sequence variants TTCAGG and TTTCAGG (Tran et al. 2015) and in *Ascaris lumbricoides* (Linnaeus, 1758) – TTAGGC (Müller et al. 1991). In general, however, it is assumed that this sequence in vertebrates consists of (TTAGGG)_n_ tandem repeats (Moyzis et al. 1988), (TTAGG)_n_ in arthropods (Kuznetsova et al. 2015), and in most plants – (TTTAGGG)_n_ (Richards and Ausubel 1988). The telomere sequence is usually very homogeneous, particularly in contrast to the subtelomeric sequences constituting a border region between the telomere and the region where genes are located. The subtelomeric regions include a fragment of about 500 kb (Macina et al. 1994) and similarly as telomeres, it consists of repetitive DNA sequences. However, the presence of genes and CpG islands has not been found in telomeres, while the subtelomers are characterized by the presence of a small number of genes and CpG islands (Blasco 2007). The common feature of the subtelomeric regions of various eukaryotic organisms is the presence of long arrays of tandem repetitive (TR) sequences or duplicated DNA fragments, which also include telomeric sequence motifs (Torres et al. 2011).

In mammals, the DNA stretch comprising a telomere is terminated with single-stranded free G-overhangs of varying, species-specific length (Kazda et al. 2012). G-overhangs are important for telomere maintenance, acting as a primer for telomerase (Lingner and Cech 1996). These 3’ ends form a spatial structure called the G-quadruplex (G4-DNA), which protects the telomere from exonucleases, thereby protecting the DNA strand against degradation (Sen and Gilbert 1988), and also inhibits telomerase activity (Zahler et al. 1991).

**Figure 3.**
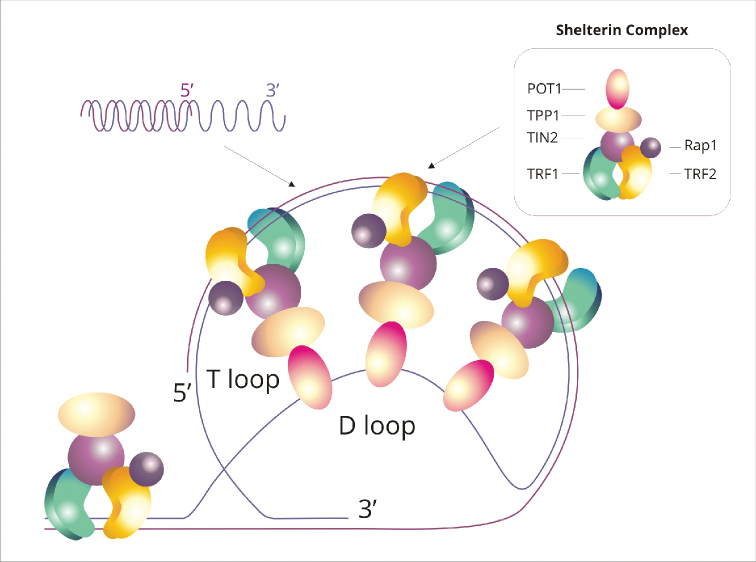
Telomere structure in mammals; T-loop and D-loop are presented together with schematic representation of the shelterin complex on telomeric DNA. The shelterin complex consists of six proteins: TRF1 and TRF2 (telomere repeat-binding factor 1 and 2), RAP1 (repressor/activator protein 1), TIN2 (TRF1-interacting nuclear factor 2), TPP1 (TINT1/PTOP/PIP1 protein) and POT1 (protection of telomeres 1).

Telomeric chromatin has a typical organization, forming the nucleosome fiber at the basal level. This structure may be different only in regions where there are telomere-specific proteins (Pisano et al. 2007). Telomere structure is formed with the participation of a protein complex called shelterin (Fig. 3). The complex consists of six proteins: TRF1 and TRF2 (telomere repeat-binding factor 1 and 2) (Zhong et al. 1992, Chong et al. 1995, Bilaud et al. 1997), RAP1 (repressor/activator protein 1), TIN2 (TRF1-interacting nuclear factor 2) (Kim et al. 1999, Li et al. 2000), TPP1 (TINT1/PTOP/PIP1 protein) (Houghtaling et al. 2004) and POT1 (protection of telomeres 1) (Baumann and Cech 2001). TRF1 and TRF2 proteins bind to telomere double-stranded DNA, while other proteins stabilize the structure of the shelterin complex. The interaction between telomere DNA and shelterin proteins first of all protects and stabilizes telomere structure, and secondly, regulates the access of proteins involved in DNA repair and elongation (de Lange 2005). Double-stranded telomeric sequence, due to interactions with shelterin proteins, folds and closes forming a larger T-loop. In turn, the free 3’ overhang at the end of the chromosome in the T-loop binds to the double-stranded telomere fragment to form a smaller D-loop. It has been found that the T-loops are characteristic of eukaryotic organism telomeres, although it is not certain whether they are present in all of them (de Lange 2004).

*D. melanogaster* telomeres have yet another structure. Three following retrotransposons have been identified in the telomere sequence: HeT-A, TART and TAHRE (HTT). At the ends of telomeres, there are numerous copies of HTT retrotransposon, while in the most proximal region, there are sequences called TAS (telomere associated sequence). The ends of telomeres are protected and stabilized by a protein complex. An important role is played by the heterochromatin 1 (HP1) protein, which binds to dimethyl lysine 9 in histone H3 (H3K9me2) (Vermaak and Malik 2009). Its absence contributes to the fusion of *Drosophila* chromosomes (Fanti et al. 1998).

In plants, telomeres are usually several kbs in size (*A. thaliana* – 2–9 kb), although they may be longer in some plants, e.g. tobacco telomeres may have a size of up to 150 kb (Richards and Ausubel 1988, Fajkus et al. 1995). G-overhang size may be 20–30 nt, however, it may not be present in all telomeres (Riha et al. 2000). Studies have shown that several proteins bind to telomeric dsDNA (double stranded DNA) as well as G-rich ssDNA (*single stranded DNA*), but they are not fully characterized. Two proteins are known that bind to single-stranded telomeric sequences: GTBP1 (G-strand specific single stranded telomere-binding protein 1) and STEP1 (single stranded telomere-binding Protein 1) (Kwon and Chung 2004, Lee and Kim 2011). Homologs of the POT1 protein, which forms a heterodimer with the TPP1 protein have been also detected (Wang et al. 2007). Studies of the function of these proteins in *A. thaliana* showed that the POT1a homologue binds telomerase and is involved in the synthesis of telomere repeats, while the POT1b and POT1c homologs are involved in the protection of chromosome termini (Shakirov et al. 2005, Kobayashi et al. 2019). In *A. thaliana*, TRB proteins (telomere repeat-binding factors) were also identified (Mozgová et al. 2008), containing a conserved domain similar to the telobox-type Myb (short telomeric motif, Myb-related DNA-binding domain) (Peška et al. 2011), through which they bind to telomeric dsDNA. This domain is typical for mammalian TRF1 and TRF2 proteins, although differently located. In TRB proteins, it is present at the N-terminus and in TRF, at the C-terminus. In addition, TRB proteins were found to possess a histone-like domain (H1/5) that plays a role in DNA-protein reactions and interaction with POT1b (Schrumpfova et al. 2008).

## Epigenetic regulation of telomere and subtelomere regions

**Figure 4.**
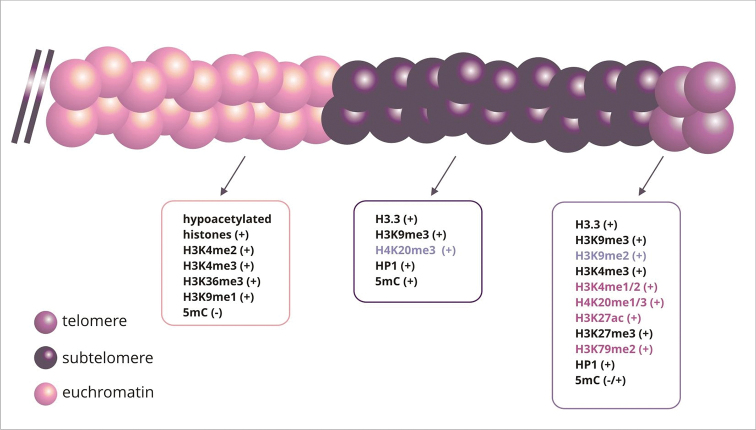
Epigenetic modifications in telomere and subtelomere chromatin and adjacent euchromatin. Epigenetic markers in telomere and subtelomere regions characteristic for both plants and animals are marked with black color, only for plants with violet colour, only for animals with rose color.

The epigenetic nature of telomeres and subtelomeres remains controversial (Vaquero-Sedas and Vega-Palas 2011, Galati et al. 2013, Ichikawa et al. 2015, Adamusová et al. 2019). In the classic model, animal and plant telomeres were interpreted as heterochromatic structures (Kavi et al. 2005, Postepska-Igielska et al. 2013). However, more and more data indicate their dual character, showing modifications of histones characteristic of both the eu- and heterochromatin fraction (Vrbsky et al. 2010) (Fig. 4). Some studies even indicate that telomeres may exhibit mainly euchromatin traits, while subtelomeres – heterochromatin features (Vaquero-Sedas et al. 2011). However, this is not definitively established, especially that even the level and occurrence of DNA methylation within telomeres remains unexplained (Blasco 2007, Vrbsky et al. 2010, Vaquero-Sedas et al. 2012, Ogrocká et al. 2014).

The variety of information regarding telomere regions may partly result from experimental limitations, but also due to the epigenetic diversity of animal (Cubiles et al. 2018) and plant cells (Majerová et al. 2014). Difficulty in determining the epigenetic state of telomeric chromatin also results from the presence of interstitial telomere repeats (ITRs) within the internal regions of chromosomes. Most of the ITRs were found within or adjacent to the constitutive heterochromatin (Meyne et al. 1990, Rodionov et al. 2002, Galkina et al. 2005, Vaquero-Sedas and Vega-Palas 2011). ITR sequences differ from typical telomere sequences in that they are heterogeneous, degenerate and contain other sequence types in addition to telomere sequence repeats (Lin and Yan 2008, Vega-Vaquero et al. 2016).

Telomeric and subtelomeric chromatin studies in mouse showed the presence of histone modifications characteristic of the heterochromatin fraction (Garcia-Cao et al. 2004, Gonzalo et al. 2006). Telomeres in vertebrates, as well as in *D. melanogaster*, are rich in H3K9me3 (Peters et al. 2001, Garcia-Cao et al. 2004). This modification is recognized by heterochromatic protein 1 (HP1), which can recruit histone methyltransferases (HMTase) such as SuM4-20h1 and SuM4-20h2, which methylate H4 at lysine 20 (H4K20me3) (Nakayama et al. 2001, Benetti et al. 2007). In telomeres, Dot1L HMTase mediates methylation of lysine 79 in H3 (H3K79me2) (Shanower et al. 2005) and methylates lysine 20 in H4 (H4K20me3) (Jones et al. 2008). In addition, histones H3 and H4 are not strongly acetylated in telomeres (Benetti et al. 2007). In human telomeres that lack SIRT6 deacetylase, a higher level of H3K9 acetylation is observed, which usually leads to telomere dysfunction (Michishita et al. 2008).

However, in mouse cells, telomeres are enriched in modifications specific to heterochromatin (H3K9me3) and euchromatin (H3K4me3). Although the H3K4me3 modification was at a lower level compared to H3K9me3 (Cao et al. 2009). ChIP-seq analysis of telomeres of various human cells has shown that they are characterized by low levels of H3K9me3, typical of heterochromatic regions, while they are enriched with euchromatin H4K20me1 and H3K27ac modifications (Rosenfeld et al. 2009, O’Sullivan et al. 2010, Cubiles et al. 2018).

Similar results were obtained in studies on plant telomeres. In Arabidopsis, heterochromatin modifications, such as H3K9me2 and H3K27me3, as well as euchromatin H3K4me3 modification have been reported (Vrbsky et al. 2010, Majerová et al. 2014, Adamusová et al. 2019). This occurrence of both heterochromatin and euchromatin modifications in the Arabidopsis telomere region was defined as the presence of an “intermediate” heterochromatin (Vrbsky et al. 2010, Majerová et al. 2014). Subsequent studies have shown that histones in telomeres have modifications typical of euchromatin, while histones within ITR regions possess modifications typical of condensed chromatin (Vaquero-Sedas et al. 2012). In the case of *Ballantinia antipoda* (Mueller, 1974), the H3K9me2 heterochromatin modification occurred mainly in telomeres, and H3K4me3 was found at a lower level, whereas only the H3K9me2 modification was present in the ITR region. Thus, it can be concluded that the chromatin of telomeres has both euchromatin and heterochromatin epigenetic markers, while the ITR regions are mainly heterochromatic (Majerová et al. 2014). In *A. thaliana* (Vrbsky et al. 2010) and *Nicotiana tabacum* (Linnaeus, 1753) telomeres, in addition to H3K9me2 and H3K4me3 modifications, the presence of H3K27me3 modifications was found, typical for heterochromatin, and it also occurs in human telomeres (Boros et al. 2014), although it is absent in mouse telomeres (Saksouk et al. 2014). Recent studies of human telomeres revealed that the PRC 2 (Polycomb 2) complex is responsible for the occurrence of H3K27me3, which affects the H3K9me3 heterochromatic modification to recruit HP1 to heterochromatin (Boros et al. 2014). It was also found that the TERRA transcript (TElomeric Repeat-containing RNA) is necessary for telomeric heterochromatin formation, the amount of modifications such as H3K9me3, H4K20me3 and H3K27me3 depends on the level of the TERRA transcript (Montero et al. 2018). It was found that lower levels of this transcript were associated with a decrease in the level of heterochromatin modifications in telomeres, H3K9m3 in particular (Deng et al. 2009).

Studies on telomere DNA methylation have not found so many discrepancies. Telomeres in mammalian cells are deprived of CpG dinucleotides, and therefore do not undergo DNA methylation (Draskovic and Londono-Vallejo 2013). Methylation studies of telomere sequences in plants have yielded conflicting results. Cytosine methylation in telomere CCCTAAA repeats was found in *A. thaliana* (Cokus et al. 2008), *N. tabacum* (Majerová et al. 2011), as well as in some other plants (Majerová et al. 2014). In turn, other studies on *A. thaliana* telomere DNA revealed low or no methylation (Vega-Vaquero et al. 2016). Detailed studies have shown that ITR sequences and sequences at the border of the telomere/subtelomere region are characterized by high levels of cytosine methylation (Cokus et al. 2008, Vrbsky et al. 2010, Vaquero-Sedas et al. 2012, Ogrocká et al. 2014). Very low level of genomic DNA methylation caused disturbances in telomere homeostasis in *A. thaliana* (Ogrocká et al. 2014, Xie and Shippen 2018), while no such changes were observed in *N. tabacum* (Majerová et al. 2011). This shows the differences in the role of DNA methylation in the regulation of telomere homeostasis in various plants (Fojtová and Fajkus 2014, Procházková-Schrumpfová et al. 2019).

While there is great controversy about the heterochromatic nature of telomeres, most studies show that this chromatin fraction is characteristic of subtelomeric regions. In animal and human cells, the subtelomeric regions are characterized by high CpG methylation and trimethylation of lysine 9 in histone H3 (H3K9me3) (Gonzalo et al. 2006). They can have a silencing effect on the expression of adjacent genes, as well as TERRA transcription. This silencing is defined as the telomere position effect (Azzalin et al. 2007, Cubiles et al. 2018). The analysis of most plant subtelomeric regions has also shown a high level of DNA methylation (Majerová et al. 2014, Ogrocká et al. 2014).

The heterochromatic state plays an important role in telomere biology, suggesting that the integrity of the subtelomeric heterochromatin may be important for the proper functioning of telomeres. A correlation was found between changes in the level of DNA methylation in the subtelomeric region and regulation of telomere length (Garcia-Cao et al. 2004, Gonzalo et al. 2006). In Arabidopsis, the subtelomeric region regulates the telomere length homeostasis. Genome hypomethylation in *A. thaliana* caused shortening of telomeres, although it was not so extensive to lead to genomic or chromosomal instability (Fajkus et al. 1995, Ogrocká et al. 2014). It has also been shown that post-translational modifications of histones have no effect on telomere length in *N. tabacum* (Majerová et al. 2011).

In budding yeasts, heterochromatinization of the subtelomeric region positively regulates telomere length (Nislow et al. 1997). For animals the opposite is true, a decrease in the occurrence of heterochromatin markers, including DNA methylation in the subtelomeric region, correlates with telomere elongation and increased recombination (Gonzalo et al. 2006, Benetti et al. 2007, Blasco 2007, Ng et al. 2009). An example is the research by Gonzalo et al. (2006), showing elongated telomeres with reduced methylation of the subtelomeric regions. Mouse mutants lacking DNA methyltransferases DNMT1 or DNMT3A and DNMT3B have very long telomeres and exhibit ALT (alternative lengthening of telomeres) characteristics, i.e. an increased rate of T-SCE (telomeric sister chromatin exchange) and the presence of APB (ALT-associated PML body) (Gonzalo et al. 2006).

Surprisingly, different reports have indicated that the length of telomeres does not change in epigenetic mutants (Roberts et al. 2011), or shown the association of very short telomeres with hypomethylation of subtelomeric regions (Benetti et al. 2007) or global hypomethylation (Pucci et al. 2013). In addition, telomere elongation has been linked to DNMT3A targeting to subtelomeric regions, resulting in increased DNA methylation (Cubiles et al. 2018).

For a long time, telomeres were perceived as silenced, transcriptionally inactive chromosome segments. This fact is negated by the presence of telomeric RNAs containing UUAGGG repeats, called TERRA, which are transcribed from the subtelomeric regions towards the ends of the chromosome by RNA pol II in yeasts, vertebrates and plants (Azzalin et al. 2007, Luke et al. 2008). The prevalence of these transcripts suggests that this is a conservative trait associated with an important function in telomere biology (Azzalin et al. 2007, Luke et al. 2008). Two classes of TERRA promoters were found in the chromosomes, and their expression is regulated by CTCF (CCCTC-binding factor) and RAD21 cohesin (radiation-sensitive 21) (Deng et al. 2012, Porro et al. 2014, Bettin et al. 2019). Absence or decrease in RAD21 or CTCF levels results in the loss of RNA pol II binding to TERRA promoters, resulting in the reduction in TERRA expressi regions, therefore, an increase in DNA methylation in this region is associated with a decrease in the expression level (Yehezkel et al. 2008, Nergadze et al. 2009, Farnung et al. 2012). The correlation was shown between inhibition of TERRA transcription and the presence of H3K9me3, H4K20me3 and DNA methylation in telomeric and subtelomeric regions (Schoeftner and Blasco 2008, Nergadze et al. 2009, Farnung et al. 2012). Moreover, it turned out that histone acetylation and DNA hypomethylation positively affect the TERRA transcription process (Azzalin and Lingner 2008). Hypomethylation of subtelomeric sequences in mammalian cells lacking DNA methyltransferases leads to TERRA overexpression. In mouse, TERRA transcript level in cell lines with deficiency of Suv3-9h and Suv4-20h HMTase is elevated compared to wild-type mouse cells. The level of epigenetic modifications characteristic for heterochromatin also regulates TERRA transcription in yeasts (Cusanelli and Chartrand 2014). In yeast, TERRA transcripts are maintained at a low level by Rat1 (Luke et al. 2008), the Sir2/Sir3/Sir4 sirtuin complex (histone deacetylases) and Rif1 and Rif2 (Rap1-interacting factor 1 and factor 2) (Iglesias et al. 2011). These results suggest that TERRA expression depends on the epigenetic status of subtelomeres and telomeres (Iglesias et al. 2011, Arnoult et al. 2012).

Binding of the TERRA transcripts to telomeres seems to be crucial for their structure and function (Luke et al. 2008). TERRA transcripts can negatively impact telomeres elongation. TERRA is believed to bind to the telomere region and regulate the length of telomeres by negatively controlling telomerase activity (Azzalin et al. 2007, Ng et al. 2009). Cells with active telomerase show a high level of TERRA promoter methylation, in contrast to those where the presence of this enzyme is not detected (Ng et al. 2009). This is probably because TERRA telomere repeats are complementary to the RNA template of telomerase and it is inhibited by competitive base pairing (Bisoffi et al. 1998). TERRA transcripts are involved in the formation of heterochromatin at chromosome ends interacting with the HP1 proteins and H3K9me3, as well as with HMTase Suv39H1 or Polycomb Repressive Complex 2 (PRC2) (Montero et al. 2018).

The interaction of TERRA transcript with TRF1 and TRF2 proteins can facilitate the binding of TERRA to the ends of chromosomes. Due to the fact that TRF1 and TRF2 can interact with chromosomes also in different regions (especially with ITR) (Simonet et al. 2011), TERRA transcripts can also bind non-telomeric sites (Cusanelli et al. 2013). TERRA, therefore, can regulate the expression of many genes (Chu et al. 2017). TERRA forms a complex with TRF2 and ORC1 (origin recognition complex 1), which facilitates DNA replication in telomeres (Deng et al. 2009). In addition, TERRA transcription itself, by the relaxation of chromatin, influences the initiation of DNA replication in this region during the S phase of the cell cycle (Bettin et al. 2019). It has been demonstrated that the expression level of TERRA depends on the phase of the cell cycle. It is high during the transition from the G1 to S phase, it is very high in the initial S phase, while it is reduced during the transition from the G2 phase to mitosis (Porro et al. 2010).

TERRA transcripts can promote homologous recombination between telomeres by creating RNA-DNA heteroduplex (R loops) at the ends of chromosomes (Chawla and Azzalin 2008). R loops can also block replication fork progression, cause double-strand breaks, delay cell aging and maintain genomic instability (Cusanelli and Chartrand 2015, Sollier and Cimprich 2015). For example, in the cells of the ICF syndrome, no methylation of the subtelomeric DNA was found, due to mutations in the DNMT3B gene. This results in a high level of the TERRA transcript, which forms telomeric R-loops, which in turn causes telomere dysfunctions (Cubiles et al. 2018). In addition, TERRA transcripts play a role in DNA damage response (DDR) caused by dysfunctional telomeres (Cusanelli and Chartrand 2015). Decrease in TERRA levels resulting from either the action of siRNA (Deng et al. 2009) or ASO-LNA (antisense oligonucleotides – locked nucleic acid) (Chu et al. 2017) as well as their incorrect localization leads to many chromosome abnormalities. Depletion of TERRA transcripts activates DDR at the ends of the chromosomes, which leads to the formation of the “telomere dysfunction-induced foci” (TIF) (Lopez de Silanes et al. 2010). Hence, proper expression and localization of TERRA is required to maintain telomeres and chromosomal stability (reviewed in Bettin et al. 2019).

Histone substitution with their variants is another epigenetic mechanism that plays a role in the functioning of telomeres. In human and mouse cells, histone H3.3 variant was correlated with TERRA transcriptional repression in telomeres and subtelomeres (Law et al. 2010). Telomeric histone H3.3 variant is deposited through the ATRX (alpha thalassemia/mental retardation syndrome x-linked)-DAXX (death-domain associated protein) complex. The loss of the function of this complex results in the reduction of modifications characteristic of heterochromatin fractions in telomeric regions, also associated with lower H3.3 levels. It has the destabilizing effect through increased homologous recombination of telomeres, which facilitates ALT (Heaphy et al. 2011). MacroH2A1.2 histone variant involvement in ALT has also been demonstrated. MacroH2A1.2 is present in telomeres, especially in ALT cells, being a mediator of homologous recombination and response to replication stress (Kim et al. 2019). H2A.Z is another histone variant that occurs in telomeres. In *S. cerevisiae* H2A.Z variants hinder the spread of the heterochromatin (Grunstein and Gasser 2013). A strong anticorrelation was found between this histone variant deposition and DNA methylation (Zilberman et al. 2008, Kobor and Lorincz 2009). Higher levels of the histone H2A.Z variant were observed in *A. thaliana* mutants with reduced DNA methylation. Thus, it can be pointed out that H2A.Z deposition somehow protects the genome against DNA methylation (Zilberman et al. 2008). The study of the *Trypanosoma brucei* (Plimmer and Bradford, 1899) chromatin showed the presence of the H3V (histone H3 variant) protein in the telomeres. It has been found that H3V has several features common to cenH3, however, its absence does not disrupt chromosomal segregation (Lowell and Cross 2004). Another example of the histone variant is sperm-specific spH2B. This variant of H2B forms a specific complex with DNA *in vitro*, which may indicate its role in the recognition of telomeric DNA. It is also believed that this protein may be involved in the attachment of telomeres to the nuclear envelope (Gineitis et al. 2000).

## Conclusions

Centromeres and telomeres are indispensable elements of every functional chromosome in Eukaryota. Considering the conservative role, their structure should be similar, not only in the context of the DNA nucleotide sequence, but also at the level of chromatin organization. Whereas in the case of telomeres this can be seen, in centromeres the similarity is observed mainly at the level of epigenetic modifications, with a great diversity of nucleotide sequences. Although microscopic analysis indicates that they are heterochromatin elements, they should now be considered as specific regions of the so-called intermediate heterochromatin, i.e. having epigenetic features of both euchromatin and heterochromatin. Undoubtedly, epigenetic status plays an extremely important role in regulating both telomeres and centromeres. For it is the specific structure of chromatin, and not just the DNA sequence itself, that ensures the proper functioning of these regions during the entire cell cycle. Many analyses have been carried out, the results of which were often contradictory, hindering an unambiguous determination of epigenetic markers of centromeric and telomeric regions.

However, these analyses have allowed us to perceive the epigenetic nature of telomeres and centromeres as very complex systems, precisely regulated at many levels. Disorders of this regulation can lead to destabilization of the entire genome. It also turned out that adjacent regions, i.e. subtelomeres and pericentromeres, often no less important than key elements, were thought for a long time to be heterochromatin boundary areas. Currently, it seems that maintaining their epigenetic status affects the structure and functioning of telomeres and centromeres. There is a need for further research on other species that will allow better understanding of telomere and centromere regulation systems in all their complexity.

## References

[B1] Aagaard L Laible G Selenko P Schmid M Dorn R Schotta G Kuhfittig S Wolf A Lebersorger A Singh PB Reuter G Jenuwein T (1999) Functional mammalian homologues of the *Drosophila* PEV-modifier Su(var)3-9 encode centrosome-associated proteins which complex with the heterochromatin component M31. EMBO Journal 18: 1923–1938. 10.1093/emboj/18.7.1923 10202156 PMC1171278

[B2] Adamusová K Khosravi S Fujimoto S Houben A Matsunaga S Fajkus J Fojtová M (2019) Two combinatorial patterns of telomere histone marks in plants with canonical and non-canonical telomere repeats. Plant Journal. 10.1111/tpj.14653 31834959

[B3] Alcan C Ventura M Archidiacono N Rocchi M Sahinalp SC Eichler EE (2007) Organization and evolution of primate centromeric DNA from whole-genome shotgun sequence data. PLOS Computational Biology 3: 1807–1818. 10.1371/journal.pcbi.0030181 17907796 PMC1994983

[B4] Aldrup-Macdonald ME Sullivan BA (2014) The past, present, and future of human centromere genomics. Genes 5: 33–50. 10.3390/genes5010033 24683489 PMC3966626

[B5] Alfenito MR Birchler JA (1993) Molecular characterization of a maize B chromosome centric sequence. Genetics 135: 589–597. https://www.ncbi.nlm.nih.gov/pmc/articles/PMC1205658/10.1093/oxfordjournals.jhered.a111297 8244015 PMC1205658

[B6] Allshire RC Karpen GH (2008) Epigenetic regulation of centromeric chromatin: old dogs, new tricks? Nature Reviews Genetics 9(12): 923–37. 10.1038/nrg2466 PMC2586333 19002142

[B7] Allshire RC Nimmo ER Ekwal K Javerzat J-P Cranston G (1995) Mutations derepressing silent centromeric domains in fission yeast disrupt chromosome segregation. Genes and Development 9: 218–233. 10.1101/gad.9.2.218 7851795

[B8] Alonso A Fritz B Hasson D Abrusan G Cheung F Yoda K Radlwimmer B Ladurner AG Warburton PE (2007) Co-localization of CENP-C and CENP-H to discontinuous domains of CENP-A chromatin at human neocentromeres. Genome Biology 8: R148. 10.1186/gb-2007-8-7-r148 PMC2323242 17651496

[B9] Altemose N Miga KH Maggioni M Willard HF (2014) Genomic characterization of large heterochromatic gaps in the human genome assembly. PLOS Computational Biology 10: e1003628. 10.1371/journal.pcbi.1003628 PMC4022460 24831296

[B10] Amaral PP Mattick JS (2008) Noncoding RNA in development. Mammalian Genome 19: 454–492. 10.1007/s00335-008-9136-7 18839252

[B11] Ananiev EV Phillips RL Rines HW (1998) Chromosome-specific molecular organization of maize (*Zea mays* L.) centromeric regions. Proceedings of the National Academy of Sciences of the United States of America 95: 13073–13078. 10.1073/pnas.95.22.13073 9789043 PMC23713

[B12] Arnoult N Van Beneden A Decottignies A (2012) Telomere length regulates TERRA levels through increased trimethylation of telomeric H3K9 and HP1α. Nature Structural and Molecular Biology 19: 948–956. 10.1038/nsmb.2364 22922742

[B13] Artandi SE DePinho RA (2010) Telomeres and telomerase in cancer. Carcinogenesis 31: 9–18. 10.1093/carcin/bgp268 19887512 PMC3003493

[B14] Azzalin CM Lingner J (2008) Telomeres: The silence is broken. Cell Cycle 7: 1161–1165. 10.4161/cc.7.9.5836 18418035

[B15] Azzalin CM Reichenbach P Khoriauli L Giulotto E Lingner J (2007) Telomeric repeat containing RNA and RNA surveillance factors at mammalian chromosome ends. Science 318: 798–801. 10.1126/science.1147182 17916692

[B16] Baumann P Cech TR (2001) Pot1, the putative telomere end-binding protein in fission yeast and humans. Science 292: 1171–1175. 10.1126/science.1060036 11349150

[B17] Belotserkovskaya R Oh S Bondarenko VA Orphanides G Studitsky VM Reinberg D (2003) FACT Facilitates transcription-dependent nucleosome alteration. Science 301(5636): 1090–1093. 10.1126/science.1085703 12934006

[B18] Benetti R Garcia-Cao M Blasco MA (2007) Telomere length regulates the epigenetic status of mammalian telomeres and subtelomeres. Nature Genetics 39: 243–250. 10.1038/ng1952 17237781

[B19] Bergmann JH Jakubsche JN Martins NM Kagansky A Nakano M Kimura H Kelly DA Turner BM Masumoto H Larionov V Earnshaw WC (2012) Epigenetic engineering: histone H3K9 acetylation is compatible with kinetochore structure and function. Journal of Cell Science 125: 411–421. 10.1242/jcs.090639 22331359 PMC3283876

[B20] Bergmann JH Rodriguez MG Martins NM Kimura H Kelly DA Masumoto H Larionov V Jansen LE Earnshaw WC (2011) Epigenetic engineering shows H3K4me2 is required for HJURP targeting and CENP-A assembly on a synthetic human kinetochore. EMBO Journal 30: 328–40. 10.1038/emboj.2010.329 21157429 PMC3025471

[B21] Bernard P Maure JF Partridge JF Genier S Javerzat JP Allshire RC (2001) Requirement of heterochromatin for cohesion at centromeres. Science 294: 2539–2542. 10.1126/science.1064027 11598266

[B22] Bettin N Pegorar CO Cusanelli E (2019) The emerging roles of TERRA in telomere maintenance and genome stability. Cells 8: 246. 10.3390/cells8030246 PMC6468625 30875900

[B23] Bilaud T Brun C Ancelin K Koering CE Laroche T Gilson E (1997) Telomeric localization of TRF2, a novel human telobox protein. Nature Genetics 17: 236–239. 10.1038/ng1097-236 9326951

[B24] Birchler JA Han F (2009) Maize centromeres: structure, function, epigenetics. Annual Review of Genetics 43: 287–303. 10.1146/annurev-genet-102108-134834 19689211

[B25] Bisoffi M Chakerian AE Fore ML Bryant JE Hernandez JP Moyzis RK Griffith JK (1998) Inhibition of human telomerase by a retrovirus expressing telomeric antisense RNA. European Journal of Cancer 34: 1242–1249. 10.1016/S0959-8049(98)00049-5 9849487

[B26] Black BE Jansen LET Maddox PS Foltz DR Desai AB Shah JV Cleveland DW (2007) Centromere identity maintained by nucleosomes assembled with histone H3 containing the CENP-A targeting domain. Molecular Cell 25: 309–322. 10.1016/j.molcel.2006.12.018 17244537

[B27] Black EM Giunta S (2018) Repetitive fragile sites: centromere satellite DNA as a source of genome instability in human diseases. Genes 9(12): 615 10.3390/genes9120615 PMC6315641 30544645

[B28] Blasco MA (2007) The epigenetic regulation of mammalian telomeres. Nature Reviews Genetics 8: 299–309. 10.1038/nrg2047 17363977

[B29] Blower MD Sullivan BA Karpen GH (2002) Conserved organization of centromeric chromatin in flies and humans. Developmental Cell 2: 319–330. 10.1016/S1534-5807(02)00135-1 11879637 PMC3192492

[B30] Bobkov GOM Gilbert N Heun P (2018) Centromere transcription allows CENP-A to transit from chromatin association to stable incorporation. Journal of Cell Biology 217: 1957–1972. 10.1083/jcb.201611087 29626011 PMC5987708

[B31] Boldyreva LV Goncharov FP Demakova OV Zykova TY Levitsky VG Kolesnikov NN Pindyurin AV Semeshin VF Zhimulev IF (2017) Protein and genetic composition of four chromatin types in *Drosophila melanogaster* cell lines. Current Genomics 18(2): 214–226. 10.2174/1389202917666160512164913 28367077 PMC5345337

[B32] Boros J Arnoult N Stroobant V Collet J-F Decottignies A (2014) Polycomb repressive complex 2 and H3K27me3 cooperate with H3K9 methylation to maintain heterochromatin protein 1α at chromatin. Molecular and Cellular Biology 34(19): 3662–3674. 10.1128/MCB.00205-14 25047840 PMC4187721

[B33] Cam HP Sugiyama T Chen ES Chen X FitzGerald PC Grewal SIS (2005) Comprehensive analysis of heterochromatin- and RNAi-mediated epigenetic control of the fission yeast genome. Nature Genetics 37: 809–819. 10.1038/ng1602 15976807

[B34] Cao F Li X Hiew S Brady H Liu Y Dou Y (2009) Dicer independent small RNAs associate with telomeric heterochromatin. RNA 15: 1274–1281. 10.1261/rna.1423309 19460867 PMC2704082

[B35] Carone DM Longo MS Ferreri GC Hall L Harris M Shook N Bulazel KV Carone BR Obergfell C O’Neill MJ O’Neill RJ (2009) A new class of retroviral and satellite encoded small RNAs emanates from mammalian centromeres. Chromosoma 118(1): 113–125. 10.1007/s00412-008-0181-5 18839199

[B36] Carroll CW Silva MCC Godek KM Jansen LE Straight AF (2009) Centromere assembly requires the direct recognition of CENP-A nucleosomes by CENP-N. Nature Cell Biology 11: 896–902. 10.1038/ncb1899 19543270 PMC2704923

[B37] Chan FL Wong LH (2012) Transcription in the maintenance of centromere chromatin identity. Nucleic Acids Research 40: 11178–11188. 10.1093/nar/gks921 23066104 PMC3526279

[B38] Chan K-L Roig MB Hu B Beckouët F Metson J Nasmyth K (2012) Cohesin’s DNA exit gate is distinct from its entrance gate and is regulated by acetylation. Cell 150: 961–974. 10.1016/j.cell.2012.07.028 22901742 PMC3485559

[B39] Charlesworth B Sniegowski P Stephan W (1994) The evolutionary dynamics of repetitive DNA in eukaryotes. Nature 371: 215–220. 10.1038/371215a0 8078581

[B40] Chawla R Azzalin CM (2008) The telomeric transcriptome and SMG proteins at the crossroads. Cytogenetic and Genome Research 122: 194–201. 10.1159/000167804 19188687

[B41] Cheng Z Dong F Langdon T Ouyang S Buell CR Gu M Blattner FR Jiang J (2002) Functional rice centromeres are marked by a satellite repeat and a centromere-specific retrotransposon. Plant Cell 14: 1691–1704. 10.1105/tpc.003079 12172016 PMC151459

[B42] Chiatante G Giannuzzi G Calabrese FM Eichler EE Ventura M (2017) Centromere destiny in dicentric chromosomes: new insights from the evolution of human chromosome 2 ancestral centromeric region. Molecular Biology and Evolution 34(7): 1669–1681. 10.1093/molbev/msx108 28333343 PMC5722054

[B43] Chong L van Steensel B Broccoli D Erdjument-Bromage H Hanish J Tempst P de Lange T (1995) A human telomeric protein. Science 270: 1663–1667. 10.1126/science.270.5242.1663 7502076

[B44] Choo KHA (2000) Centromerization. Trends Cell Biology 10: 182–188. 10.1016/S0962-8924(00)01739-6 10754560

[B45] Choo KHA (2001) Domain organization at the centromere and neocentromere. Developmental Cell 1: 165–177. 10.1016/S1534-5807(01)00028-4 11702777

[B46] Chu HP Cifuentes-Rojas C Kesner B Aeby E Lee HG Wei C Oh HJ Boukhali M Haas W Lee JT (2017) TERRA RNA Antagonizes ATRX and Protects Telomeres. Cell 170: 86–101.e16. 10.1016/j.cell.2017.06.017 PMC5552367 28666128

[B47] Chueh AC Northrop EL Brettingham-Moore KH Choo KH Wong LH (2009) LINE retrotransposon RNA is an essential structural and functional epigenetic component of a core neocentromeric chromatin. PLoS Genetics 5: e1000354. 10.1371/journal.pgen.1000354 PMC2625447 19180186

[B48] Clapier CR Cairns BR (2009) The biology of chromatin remodeling complexes. Annual Review of Biochemistry 78: 273–304. 10.1146/annurev.biochem.77.062706.153223 19355820

[B49] Cleveland DW Mao Y Sullivan KF (2003) Centromeres and kinetochores: from epigenetics to mitotic checkpoint signaling. Cell 112: 407–421. 10.1016/S0092-8674(03)00115-6 12600307

[B50] Cloix C Tutois S Yukawa Y Mathieu O Cuvillier C Espagnol MC Picard G Tourmente S (2002) Analysis of the 5S RNA pool in *Arabidopsis thaliana*: RNAs are heterogeneous and only two of the genomic 5S loci produce mature 5S RNA. Genome Research 12(1): 132–144. 10.1101/gr.181301 11779838 PMC155267

[B51] Cokus SJ Feng SH Zhang XY Chen ZG Merriman B Haudenschild CD Pradhan S Nelson SF Pellegrini M Jacobsen SE (2008) Shotgun bisulphite sequencing of the *Arabidopsis* genome reveals DNA methylation patterning. Nature 452: 215–219. 10.1038/nature06745 18278030 PMC2377394

[B52] Csink AK Henikoff S (1998) Something from nothing: the evolution and utility of satellite repeats. Trends Genetics 14(5): 200–204. 10.1016/S0168-9525(98)01444-9 9613205

[B53] Cubiles MD Barroso S Vaquero-Sedas MI Enguix A Aguilera A Vega-Palas MA (2018) Epigenetic features of human telomeres. Nucleic Acids Research 46: 2347–2355. 10.1093/nar/gky006 29361030 PMC5861411

[B54] Cusanelli E Chartrand P (2014) Telomeric noncoding RNA: Telomeric repeat-containing RNA in telomere biology. Wiley Interdisciplinary Reviews RNA 5: 407–419. 10.1002/wrna.1220 24523222

[B55] Cusanelli E Chartrand P (2015) Telomeric repeat-containing RNA TERRA: anoncoding RNA connecting telomere biology to genome integrity. Frontiers in Genetics 6 (143). 10.3389/fgene.2015.00143 PMC4396414 25926849

[B56] Cusanelli E Romero CA Chartrand P (2013) Telomeric noncoding RNA TERRA is induced by telomere shortening to nucleate telomerase molecules at short telomeres. Molecular Cell 51: 780–791. 10.1016/j.molcel.2013.08.029 24074956

[B57] De Lange T (2004) T-loops and the origin of telomeres. Nature Reviews Molecular Cell Biology 5: 323–329. 10.1038/nrm1359 15071557

[B58] De Lange T (2005) Shelterin: the protein complex that shapes and safeguards human telomeres. Genes and Development 19: 2100–2110. 10.1101/gad.1346005 16166375

[B59] Deng Y Chan SS Chang S (2008) Telomere dysfunction and tumour suppression: the senescence connection. Nature Reviews Cancer 8: 450–458. 10.1038/nrc2393 18500246 PMC3688269

[B60] Deng Z Norseen J Wiedmer A Riethman H Lieberman PM (2009) TERRA RNA binding to TRF2 facilitates heterochromatin formation and ORC recruitment at telomeres. Molecular Cell 35: 403–413. 10.1016/j.molcel.2009.06.025 19716786 PMC2749977

[B61] Deng Z Wang Z Stong N Plasschaert R Moczan A Chen HS Hu S Wikramasinghe P Davuluri RV Bartolomei MS Riethman H Lieberman PM (2012) A role for CTCF and cohesin in subtelomere chromatin organization, TERRA transcription, and telomere end protection. EMBO Journal 31: 4165–4178. 10.1038/emboj.2012.266 23010778 PMC3492729

[B62] Drané P Ouararhni K Depaux A Shuaib M Hamiche A (2010) The death-associated protein DAXX is a novel histone chaperone involved in the replication-independent deposition of H3.3. Genes and Development 24: 1253–1265. 10.1101/gad.566910 20504901 PMC2885661

[B63] Draskovic I Londono-Vallejo A (2013) Telomere recombination and alternative telomere lengthening mechanisms. Frontiers in Bioscience (Landmark Ed) 18: 1–20. 10.2741/4084 23276906

[B64] Du Y Topp CN Dawe RK (2010) DNA binding of centromere protein C (CENPC) is stabilized by single-stranded RNA. PLoS Genetic 6(2): e1000835. 10.1371/journal.pgen.1000835 PMC2816676 20140237

[B65] Duda Z Trusiak S O’Neill R (2017) Centromere transcription: means and motive. Progress in Molecular and Subcellular Biology 56: 257–281. 10.1007/978-3-319-58592-5_11 28840241

[B66] Dupont C Armant DR Brenner CA (2009) Epigenetics: definition, mechanisms and clinical perspective. Seminars in Reproductive Medicine 27(5): 351–357. 10.1055/s-0029-1237423 19711245 PMC2791696

[B67] Fachinetti D Han JS McMahon MA Ly P Abdullah A Wong AJ Cleveland DW (2015) DNA sequence-specific binding of CENP-B enhances the fidelity of human centromere function. Developmental Cell 33(3): 314–327. 10.1016/j.devcel.2015.03.020 25942623 PMC4421092

[B68] Fajkus J Kovarik A Kralovics R Bezdek M (1995) Organization of telomeric and subtelomeric chromatin in the higher plant *Nicotiana tabacum*. Molecular Genetics and Genomics 247: 633–638. 10.1007/BF00290355 7603443

[B69] Fajkus P Peska V Sitova Z Fulneckova J Dvorackova M Gogela R Sykorova E Hapala J Fajkus J (2016) *Allium* telomeres unmasked: the unusual telomeric sequence (CTCGGTTATGGG)n is synthesized by telomerase. Plant Journal 85: 337–347. 10.1111/tpj.13115 26716914

[B70] Falk M Feodorova Y Naumova N Imakaev M Lajoie BR Leonhardt H Joffe B Dekker J Fudenberg G Solovei I Mirny L (2019) Heterochromatin drives compartmentalization of inverted and conventional nuclei. Nature 570: 395–399. 10.1038/s41586-019-1275-3 31168090 PMC7206897

[B71] Fanti L Giovinazzo G Berloco M Pimpinelli S (1998) The heterochromatin protein 1 prevents telomere fusions in *Drosophila*. Molecular Cell 2(5): 257–238. 10.1016/S1097-2765(00)80152-5 9844626

[B72] Farnung BO Brun CM Arora R Lorenzi LE Azzalin CM (2012) Telomerase efficiently elongates highly transcribing telomeres in human cancer cells. PLoS ONE 7:e35714. 10.1371/journal.pone.0035714 PMC3338753 22558207

[B73] Feng C Yuan J Bai H Liu Y Su H Liu Y Shi L Gao Z Birchler JA Han F (2019) The deposition of cenH3 in maize is stringently regulated. The Plant Journal. 10.1111/tpj.14606 31713923

[B74] Feng W Hale CJ Over RS Cokus SJ Jacobsen SE Michaels SD (2017) Large-scale heterochromatin remodeling linked to overreplication-associated DNA damage. Proceedings of the National Academy of Sciences of the United States of America 114(2): 406–411. 10.1073/pnas.1619774114 28028228 PMC5240675

[B75] Feng S Cocus SJ Zhang X Chen PY Bostick M Goll MG Hetzel J Jain J Strauss SH Halpern ME Ukomadu C Sadler KC Pradhan S Pellegrini M Jacobsen SE (2010) Conservation and divergence of methylation patterning in plants and animals. Proceedings of the National Academy of Sciences of the United States of America 107: 8689–8694. 10.1073/pnas.1002720107 20395551 PMC2889301

[B76] Ferri F Bouzinba-Segard H Velasco G Hubé F Francastel C (2009) Non-coding murine centromeric transcripts associate with and potentiate Aurora B kinase. Nucleic Acids Research 37: 5071–5080. 10.1093/nar/gkp529 19542185 PMC2731909

[B77] Filion GJ van Bemmel JG Braunschweig U Talhout W Kind J Ward LD Brugman W de Castro IJ Kerkhoven RM Bussemaker HJ van Steensel B (2010) Systematic protein location mapping reveals five principal chromatin types in Drosophila cells. Cell 143(2): 212–224. 10.1016/j.cell.2010.09.009 20888037 PMC3119929

[B78] Flemming W (1882) Zellsubstanz, Kern und Zelltheilung. FCW, Vogel. 10.5962/bhl.title.168645

[B79] Fojtová M Fajkus J (2014) Epigenetic regulation of telomere maintenance. Cytogenetics Genome Research 143(1-3): 125–35. 10.1159/000360775 24714070

[B80] Folco HD Pidoux AL Urano T Allshire RC (2008) Heterochromatin and RNAi are required to establish CENP-A chromatin at centromeres. Science 319: 94–97. 10.1126/science.1150944 18174443 PMC2586718

[B81] Foltz DR Jansen LET Black BE Bailey AO Yates 3rd JR Cleveland DW (2006) The human CENP-A centromeric nucleosome-associated complex. Nature Cell Biology 8: 458–469. 10.1038/ncb1397 16622419

[B82] Foltz DR Jansen LE Bailey AO Yates JR 3rd Bassett EA Wood S Black BE Cleveland DW (2009) Centromere-specific assembly of CENP-a nucleosomes is mediated by HJURP. Cell 137(3): 472–84. 10.1016/j.cell.2009.02.039 19410544 PMC2747366

[B83] Fu S Lv Z Gao Z Wu H Pang J Zhang B Dong Q Guo X Wang XJ Birchler JA Han F (2013) *De novo* centromere formation on a chromosome fragment in maize. Proceedings of the National Academy of Sciences of the United States of America 110: 6033–6036. 10.1073/pnas.1303944110 23530217 PMC3625319

[B84] Furuyama S Biggins S (2007) Centromere identity is specified by a single centromeric nucleosome in budding yeast. Proceedings of the National Academy of Sciences of the United States of America 104: 14706–14711. 10.1073/pnas.0706985104 17804787 PMC1976213

[B85] Galati A Micheli E Cacchione S (2013) Chromatin structure in telomere dynamics. Frontiers in Oncology 3: 46. 10.3389/fonc.2013.00046 PMC3590461 23471416

[B86] Galkina S Lukina N Zakharova K Rodionov AV (2005) Interstitial (TTAGGG)(n) sequences are not hot spots of recombination in the chicken lampbrush macrochromosomes 1–3. Chromosome Research 13: 551–557. 10.1007/s10577-005-0980-y 16170619

[B87] Garcia-Cao M O’Sullivan R Peters AH Jenuwein T Blasco MA (2004) Epigenetic regulation of telomere length in mammalian cells by the Suv39h1 and Suv39h2 histone methyltransferases. Nature Genetics 36: 94–99. 10.1038/ng1278 14702045

[B88] Gent JI Dawe RK (2012) RNA as a structural and regulatory component of the centromere. Annual Review of Genetics 46: 443–53. 10.1146/annurev-genet-110711-155419 22974300

[B89] Gieni RS Chan GKT Hendzel MJ (2008) Epigenetics Regulate Centromere Formation and Kinetochore Function. Journal of Cellular Biochemistry 104(6): 2027–2039. 10.1002/jcb.21767 18404676

[B90] Gineitis AA Zalenskaya IA Yau PM Bradbury EM Zalensky AO (2000) Human sperm telomere-binding complex involves histone H2B and secures telomere membrane attachment. Journal of Cell Biology 151(7): 1591–1598. 10.1083/jcb.151.7.1591 11134086 PMC2150669

[B91] Gong Z Wu Y Koblízková A Torres GA Wang K Iovene M Neumann P Zhang W Novák P Buell CR Macas J Jiang J (2012) Repeatless and repeat-based centromeres in potato: implications for centromere evolution. Plant Cell 24: 3559–3574. 10.1105/tpc.112.100511 22968715 PMC3480287

[B92] González-Barrios R Soto-Reyes E Herrera LA (2012) Assembling pieces of the centromere epigenetics puzzle. Epigenetics 7: 3–13. 10.4161/epi.7.1.18504 22207360 PMC3329500

[B93] Gonzalo S Jaco I Fraga MF Chen T Li E Esteller M Blasco MA (2006) DNA methyltransferases control telomere length and telomere recombination in mammalian cells. Nature Cell Biology 8: 416–424. 10.1038/ncb1386 16565708

[B94] Grewal SIS Jia S (2007) Heterochromatin revisited. Nature Reviews Genetics 8: 35–46. 10.1038/nrg2008 17173056

[B95] Grimes BR Rhoades AA Willard HF (2002) α-Satellite DNA and vector composition influence rates of human artificial chromosome formation. Molecular Therapy 5: 798–805. 10.1006/mthe.2002.0612 12027565

[B96] Grunstein M Gasser SM (2013) Epigenetics in *Saccharomyces cerevisiae* Cold Spring Harb Perspectives in Biology 5(7): a017491. 10.1101/cshperspect.a017491 PMC3685889 23818500

[B97] Guenatri M Bailly D Maison C Almouzni G (2004) Mouse centric and pericentric satellite repeats form distinct functional heterochromatin. Journal of Cell Biology 166: 493–505. 10.1083/jcb.200403109 15302854 PMC2172221

[B98] Han F Lamb JC Birchler JA (2006) High frequency of centromere inactivation resulting in stable dicentric chromosomes of maize. Proceedings of the National Academy of Sciences of the United States of America 103: 3238–3243. 10.1073/pnas.0509650103 16492777 PMC1413895

[B99] Han Y Zhang Z Liu C Liu J Huang S Jiang J Jin W (2009) Centromere repositioning in cucurbit species: implication of the genomic impact from centromere activation and inactivation. Proceedings of the National Academy of Sciences of the United States of America 106(35): 14937–14941. 10.1073/pnas.0904833106 19706458 PMC2736423

[B100] He Q Cai Z Hu T Liu H Bao C Mao W Jin W (2015) Repetitive sequence analysis and karyotyping reveals centromere-associated DNA sequences in radish (*Raphanus sativus* L.). BMC Plant Biol 15:105. 10.1186/s12870-015-0480-y PMC4417506 25928652

[B101] Heaphy CM de Wilde RF Jiao Y Klein AP Edil BH Shi C Bettegowda C Rodriguez FJ Eberhart CG Hebbar S Offerhaus GJ McLendon R Rasheed BA He Y Yan H Bigner DD Oba-Shinjo SM Marie SK Riggins GJ Kinzler KW Vogelstein B Hruban RH Maitra A Papadopoulos N Meeker AK (2011) Altered telomeres in tumors with ATRX and DAXX mutations. Science 333: 425. 10.1126/science.1207313 PMC3174141 21719641

[B102] Heckmann S Jankowska M Schubert V Kumke K Ma W Houben A (2014) Alternative meiotic chromatid segregation in the holocentric plant *Luzula elegans* Nature Communications 5: 4979. 10.1038/ncomms5979 PMC4214429 25296379

[B103] Heckmann S Macas J Kumke K Fuchs J Schubert V Ma L Novák P Neumann P Taudien S Platzer M Houben A (2013) The holocentric species *Luzula elegans* shows interplay between centromere and large-scale genome organization. Plant Journal 73: 555–565. 10.1111/tpj.12054 23078243

[B104] Henikoff S Smith MM (2015) Histone variants and epigenetics. Cold Spring Harbor Perspectives in Biology 7(1): a019364. 10.1101/cshperspect.a019364 PMC4292162 25561719

[B105] Henikoff S Ahmad K Malik HS (2001) The centromere paradox: stable inheritance with rapidly evolving DNA. Science 293: 1098–1102. 10.1126/science.1062939 11498581

[B106] Henikoff JG Thakur J Kasinathan S Henikoff S (2015) A unique chromatin complex occupies young α-satellitearrays of human centromeres. Science Advance 1(1): e1400234. 10.1126/sciadv.1400234 PMC4410388 25927077

[B107] Hetrr E Allis CD (2005) RNA meets chromatin. Genes and Development 19: 1635–1655. 10.1101/gad.1324305 16024654

[B108] Hori T Shang W-H Toyoda A Misu S Monma N Ikeo K Molina O Vargiu G Fujiyama A Kimura H Earnshaw WC Fukagawa T (2014) Histone H4 Lys 20 monomethylation of the CENP-A nucleosome is essential for kinetochore assembly. Developmental Cell 29: 740–749. 10.1016/j.devcel.2014.05.001 24960696 PMC4081567

[B109] Houben A Schroeder-Reiter E Nagaki K Nasuda S Wanner G Murata M Endo TR (2007) cenH3 interacts with the centromeric retrotransposon cereba and GC-rich satellites and locates to centromeric substructures in barley. Chromosoma 116(3): 275–83. 10.1007/s00412-007-0102-z 17483978

[B110] Houghtaling BR Cuttonaro L Chang W Smith S (2004) A dynamic molecular link between the telomere length regulator TRF1 and the chromosome end protector TRF2. Current Biology 14: 1621–1631. 10.1016/j.cub.2004.08.052 15380063

[B111] Ichikawa Y Nishimura Y Kurumizaka H Shimizu M (2015) Nucleosome organization and chromatin dynamics in telomeres. Biomolecular Concepts 6: 67–75. 10.1515/bmc-2014-0035 25720088

[B112] Ichikawa K Tomioka S Suzuki Y Nakamura R Doi K Yoshimura J Kumagai M Inoue Y Uchida Y Irie N Takeda H Morishita S (2017) Centromere evolution and CpG methylation during vertebrate speciation. Nature Communications 8: 1833. 10.1038/s41467-017-01982-7 PMC5705604 29184138

[B113] Iglesias N Redon S Pfeiffer V Dees M Lingner J Luke B (2011) Subtelomeric repetitive elements determine TERRA regulation by Rap1/Rif and Rap1/Sir complexes in yeast. EMBO Reports 12: 587–593. 10.1038/embor.2011.73 21525956 PMC3128280

[B114] Ishii K Ogiyama Y Chikashige Y Soejima S Masuda F Kakuma T Hiraoka Y Takahashi K (2008) Heterochromatin integrity affects chromosome reorganization after centromere dysfunction. Science 321: 1088–1091. 10.1126/science.1158699 18719285

[B115] Jacob Y Stroud H Leblanc C Feng S Zhuo L Caro E Hassel C Gutierrez C Michaels SD Jacobsen SE (2010) Regulation of heterochromatic DNA replication by histone H3 lysine 27 methyltransferases. Nature 466: 987–991. 10.1038/nature09290 20631708 PMC2964344

[B116] Jamai A Puglisi A Strubin M (2009) Histone chaperone Spt16 promotes redeposition of the original H3–H4 histones evicted by elongating RNA polymerase. Molecular Cell 35: 377–383. 10.1016/j.molcel.2009.07.001 19683500

[B117] Jiang J (2013) Centromere evolution. In: Jiang J Birchler JA (Eds) Plant centromere biology.Wiley, New Jersey, 159–168. 10.1002/9781118525715.ch12

[B118] Jiang J Birchler JA Parrott WA Dawe RK (2003) A molecular view of plant centromeres. Trends in Plant Science 8(12): 570–575. 10.1016/j.tplants.2003.10.011 14659705

[B119] Jin B Tao Q Peng J Soo HM Wu W Ying J Fields CR Delmas AL Liu X Qiu J Robertson KD (2008) DNA methyltransferase 3B (DNMT3B) mutations in ICF syndrome lead to altered epigenetic modifications and aberrant expression of genes regulating development, neurogenesis and immune function. Human Molecular Genetics 17(5): 690–709. 10.1093/hmg/ddm341 18029387

[B120] Jin W Lamb JC Vega JM Dawe RK Birchler JA Jiang J (2005) Molecular and functional dissection of the maize B chromosome centromere. Plant Cell 17: 1412–1423. 10.1105/tpc.104.030643 15805482 PMC1091764

[B121] John RM Rougeulle C (2018) Developmental epigenetics: phenotype and the flexible epigenome. Frontiers in Cell and Developmental Biology 6: 130. 10.3389/fcell.2018.00130 PMC6193064 30364270

[B122] Jones B Su H Bhat A Lei H Bajko J Hevi S Baltus GA Kadam S Zhai H Valdez R Gonzalo S Zhang Y Li E Chen T (2008) The histone H3K79 methyltransferase Dot1L is essential for mammalian development and heterochromatin structure. PLoS Genetics 4:e1000190. 10.1371/journal.pgen.1000190 PMC2527135 18787701

[B123] Kabesch M Michel S Tost J (2010) Epigenetic mechanisms and the relationship to childhood asthma. European Respiratory Journal 36(4): 950–961. 10.1183/09031936.00019310 20889464

[B124] Kagansky A Folco HD Almeida R Pidoux AL Boukaba A Simmer F Urano T Hamilton GL Allshire RC (2009) Synthetic heterochromatin bypasses RNAi and centromeric repeats to establish functional centromeres. Science 324: 1716–1719. 10.1126/science.1172026 19556509 PMC2949999

[B125] Kang H Wu D Fan T Zh J (2020) Activities of chromatin remodeling factors and histone chaperones and their effects in root apical meristem development. International Journal of Molecular Sciences 21: 771. 10.3390/ijms21030771 PMC7038114 31991579

[B126] Kang J Chaudhary J Dong H Kim S Brautigam CA Yu H (2011) Mitotic centromeric targeting of HP1 and its binding to Sgo1 are dispensable for sister-chromatid cohesion in human cells. Molecular Biology of the Cell 22(8): 1181–1190. 10.1091/mbc.e11-01-0009 21346195 PMC3078076

[B127] Kato H Jiang J Zhou BR Rozendaal M Feng H Ghirlando R Xiao TS Straight AF Bai Y (2013) A conserved mechanism for centromeric nucleosome recognition by centromere protein CENP-C. Science 340(6136): 1110–1113. 10.1126/science.1235532 23723239 PMC3763809

[B128] Kavi HH Fernandez HR Xie W Birchler JA (2005) RNA silencing in *Drosophila*. FEBS Letters 579(26): 5940–5949. 10.1016/j.febslet.2005.08.069 16198344

[B129] Kawashima SA Yamagishi Y Honda T Ishiguro K Watanabe Y (2010) Phosphorylation of H2A by Bub1 prevents chromosomal instability through localizing shugoshin. Science 327: 172–177. 10.1126/science.1180189 19965387

[B130] Kazda A Zellinger B Rössler M Derboven E Kusenda B Riha K (2012) Chromosome end protection by blunt-ended telomeres. Genes and Development 26(15): 1703–1713. 10.1101/gad.194944.112 22810623 PMC3418588

[B131] Kim IS Lee M Park KC Jeon Y Park JH Hwang EJ Jeon TI Ko S Lee H Baek SH Kim KI (2012) Roles of Mis18α in epigenetic regulation of centromeric chromatin and CENP-A loading. Molecular Cell 46: 260–273. 10.1016/j.molcel.2012.03.021 22516971

[B132] Kim J Sun C Tran AD Chin PJ Ruiz PD Wang K Gibbons RJ Gamble MJ Liu Y Oberdoerffer P (2019) The macroH2A1.2 histone variant links ATRX loss to alternative telomere lengthening. Nature Structural and Molecular Biology 26(3): 213–219. 10.1038/s41594-019-0192-3 PMC6537592 30833786

[B133] Kim SH Kaminker P Campisi J (1999) TIN2, a new regulator of telomere length in human cells. Nature Genetics 23: 405–412. 10.1038/70508 10581025 PMC4940194

[B134] Kobayashi CR Castillo-González C Survotseva Y Canal E Nelson ADL Shippen DE (2019) Recent emergence and extinction of the protection of telomeres 1c gene in *Arabidopsis thaliana*. Plant Cell Reports 38: 1081–1097. 10.1007/s00299-019-02427-9 31134349 PMC6708462

[B135] Kobor MS Lorincz MC (2009) H2A.Z and DNA methylation: Irreconcilable differences. Trends in Biochemical Science 34: 158–161. 10.1016/j.tibs.2008.12.006 19282182

[B136] Koo DH Han F Birchler JA Jiang J (2011) Distinct DNA methylation patterns associated with active and inactive centromeres of the maize B chromosome. Genome Research 21: 908–914. 10.1101/gr.116202.110 21518739 PMC3106323

[B137] Kumar S Chinnusamy V Mohapatra T (2018) Epigenetics of modified DNA bases: 5-methylcytosine and beyond. Frontiers in Genetics 9: 640. 10.3389/fgene.2018.00640 PMC6305559 30619465

[B138] Kurokawa R Rosenfeld MG Glass CK (2009) Transcriptional regulation through noncoding RNAs and epigenetic modifications. RNA Biology 6: 233–236. 10.4161/rna.6.3.8329 19411842

[B139] Kursel LE Malik HS (2016) Centromeres. Current Biology 26(12): R487-R490. 10.1016/j.cub.2016.05.031 27326706

[B140] Kuznetsova VG Grozeva SM Hartung V Anokhin BA (2015) First evidence for (TTAGG)n telomeric sequence and sex chromosome post-reduction in *Coleorrhyncha* (*Insecta*, *Hemiptera*). Comparative Cytogenetics 9(4): 523–532. 10.3897/CompCytogen.v9i4.5609 26753072 PMC4698568

[B141] Kwon C Chung IK (2004) Interaction of an *Arabidopsis* RNA‐binding protein with plant single‐stranded telomeric DNA modulates telomerase activity. Journal of Biological Chemistry 279: 12812–12818. 10.1074/jbc.M312011200 14703514

[B142] Lamb JC Kato A Birchler JA (2005) Sequences associated with A chromosome centromeres are present throughout the maize B chromosome. Chromosoma 113: 337–349. 10.1007/s00412-004-0319-z 15586285

[B143] Law MJ Lower KM Voon HP Hughes JR Garrick D Viprakasit V Mitson M De Gobbi M Marra M Morris A Abbott A Wilder SP Taylor S Santos GM Cross J Ayyub H Jones S Ragoussis J Rhodes D Dunham I Higgs DR Gibbons RJ. (2010) ATR-X syndrome protein targets tandem repeats and influences allele-specific expression in a size-dependent manner. Cell 143: 367–378. 10.1016/j.cell.2010.09.023 21029860

[B144] Lee HR Zhang W Langdon T Jin W Yan H Cheng Z Jiang J (2005) Chromatin immunoprecipitation cloning reveals rapid evolutionary patterns of centromeric DNA in *Oryza* species. Proceedings of the National Academy of Sciences of the United States of America 102: 11793–11798. 10.1073/pnas.0503863102 PMC1187982 16040802

[B145] Lee YW Kim WT (2011) Roles of NtGTBP1 in telomere stability. Plant Signaling and Behavior 6: 523–525. 10.4161/psb.6.4.14749 21474994 PMC3142381

[B146] Lermontova I Sandmann M Demidov D (2014) Centromeres and kinetochores of Brassicaceae. Chromosome Research 22: 135–152. 10.1007/s10577-014-9422-z 24801345

[B147] Li B Oestreich S de Lange T (2000) Identification of human Rap1: Implications for telomere evolution. Cell 101: 471–483. 10.1016/S0092-8674(00)80858-2 10850490

[B148] Lin KW Yan J (2008) Endings in the middle: current knowledge of interstitial telomeric sequences. Mutation Research 658: 95–110. 10.1016/j.mrrev.2007.08.006 17921045

[B149] Lingner J Cech TR (1996) Purification of telomerase from *Euplotes aediculatus*: requirement of a primer 3′ overhang. Proceedings of the National Academy of Sciences of the United States of America 93: 10712–10717. 10.1073/pnas.93.20.10712 8855245 PMC38220

[B150] Lippman Z Martienssen R (2004) The role of RNA interference in heterochromatic silencing. Nature 431: 364–370. 10.1038/nature02875 15372044

[B151] Liu Y Su H Zhang J Liu Y Han F Birchler JA (2015) Dynamic epigenetic states of maize centromeres. Frontiers in Plant Science 6: 904. 10.3389/fpls.2015.00904 PMC4620398 26579154

[B152] Lopez de Silanes I Stagno d’Alcontres M Blasco MA (2010) TERRA transcripts are bound by a complex array of RNA-binding proteins. Nature Communications 1: 33. 10.1038/ncomms1032 20975687

[B153] Lowell JE Cross GAM (2004) A variant histone H3 is enriched at telomeres in *Trypanosoma brucei*. Journal of Cell Science 117: 5937–5947. 10.1242/jcs.01515 15522895

[B154] Luger K Mader AW Richmond RK Sargent DF Richmond TJ (1997) Crystal structure of the nucleosome core particle at 2.8 A resolution. Nature 389: 251–260. 10.1038/38444 9305837

[B155] Luke B Panza A Redon S Iglesias N Li Z Lingner J (2008) The Rat1p 5′ to 3′ exonuclease degrades telomeric repeat-containing RNA and promotes telomere elongation in *Saccharomyces cerevisiae*. Molecular Cell 32: 465–477. 10.1016/j.molcel.2008.10.019 19026778

[B156] Lukhtanov VA Dincă V Friberg M Šíchová J Olofsson M Vila R Marec F Wiklund C (2018) Versatility of multivalent orientation, inverted meiosis, and rescued fitness in holocentric chromosomal hybrids. Proceedings of the National Academy of Sciences of the United States of America 115(41): E9610-E9619. 10.1073/pnas.1802610115 PMC6187165 30266792

[B157] Macas J Neumann P Navrátilová A (2007) Repetitive DNA in the pea (*Pisum sativum* L.) genome: comprehensive characterization using 454 sequencing and comparison to soybean and *Medicago truncatula* BMC Genomics 8:427. 10.1186/1471-2164-8-427 PMC2206039 18031571

[B158] Macina RA Negorev DG Spais C Ruthig LA Hu XL Riethman HC (1994) Sequence organization of the human chromosome 2q telomere. Human Molecular Genetics 3: 1847–1853. 10.1093/hmg/3.10.1847 7545974

[B159] Maison C Bailly D Peters AHFM Quivy JP Roche D Taddei A Lachner M Jenuwein T Almouzni G (2002) Higher-order structure in pericentric heterochromatin involves a distinct pattern of histone modification and an RNA component. Nature Genetics 30: 329–334. 10.1038/ng843 11850619

[B160] Majerová E Fojtova M Mozgova I Bittova M Fajkus J (2011) Hypomethylating drugs efficiently decrease cytosine methylation in telomeric DNA and activate telomerase without affecting telomere lengths in tobacco cells. Plant Molecular Biology 77: 371–380. 10.1007/s11103-011-9816-7 21866390

[B161] Majerová E Mandáková T Vu GT Fajkus J Lysak MA Fojtová M (2014) Chromatin features of plant telomeric sequences at terminal vs. internal positions. Frontiers in Plant Science 5: 593. 10.3389/fpls.2014.00593 PMC4219495 25408695

[B162] Malik HS Henikoff S (2003) Phylogenomics of the nucleosome. Nature Structural and Molecular Biology 10: 882–891. 10.1038/nsb996 14583738

[B163] Malik HS Henikoff S (2009) Major evolutionary transitions in centromere complexity. Cell 138: 1067–1082. 10.1016/j.cell.2009.08.036 19766562

[B164] Mandrioli M Manicardi GC (2012) Unlocking holocentric chromosomes: new perspectives from comparative and functional genomics? Current Genomics 13: 343–349. 10.2174/138920212801619250 PMC3401891 23372420

[B165] Marión RM Blasco MA (2010) Telomeres and telomerase in adult stem cells and pluripotent embryonic stem cells. Advancesin Experimental Medicine and Biology 695: 118–131. 10.1007/978-1-4419-7037-4_9 21222203

[B166] Marshall OJ Chueh AC Wong LH Choo KHA (2008) Neocentromeres: new insights into centromere structure, disease development, and karyotype evolution. American Journal of Human Genetics 82: 261–282. 10.1016/j.ajhg.2007.11.009 18252209 PMC2427194

[B167] Masumoto H Yoda K Ikeno M Kitagawa K Muro Y Okazaki T (1993) Properties of CENP-B and its target sequence in a satellite DNA. In: Vig BK (Eds) Chromosome Segregation and Aneuploidy.NATO ASI Series (Series H: Cell Biology), Springer, Berlin, Heidelberg, 31–43. 10.1007/978-3-642-84938-1_3

[B168] Masumoto H Nakano M Ohzeki J (2004) The role of CENP-B and alphasatellite DNA: *de novo* assembly and epigenetic maintenance of human centromeres. Chromosome Research 12: 543–556. 10.1023/B:CHRO.0000036593.72788.99 15289662

[B169] May BP Lippman ZB Fang Y Spector DL Martienssen RA (2005) Differential regulation of strand-specific transcripts from *Arabidopsis* centromeric satellite repeats. PLoS Genetetics 1: e79. 10.1371/journal.pgen.0010079 PMC1317654 16389298

[B170] Melters DP Paliulis LV Korf IF Chan SW (2012) Holocentric chromosomes: convergent evolution, meiotic adaptations, and genomic analysis. Chromosome Research 20: 579–593. 10.1007/s10577-012-9292-1 22766638

[B171] Melters DP Bradnam KR Young HA Telis N May MR Ruby JG Sebra R Peluso P Eid J Rank D Garcia JF DeRisi JL Smith T Tobias C Ross-Ibarra J Korf I Chan SWL (2013) Comparative analysis of tandem repeats from hundreds of species reveals unique insights into centromere evolution. Genome Biology 14: R10. 10.1186/gb-2013-14-1-r10 PMC4053949 23363705

[B172] Meyne J Baker RJ Hobart HH Hsu TC Ryder OA Ward OG Wiley JE Wurster-Hill DH Yates TL Moyzis RK (1990) Distribution of non-telomeric sites of the (TTAGGG)n telomeric sequence in vertebrate chromosomes. Chromosoma 99(1): 3–10. 10.1007/BF01737283 2340757

[B173] Michishita E McCord RA Berber E Kioi M Padilla-Nash H Damian M Cheung P Kusumoto R Kawahara TL Barrett JC Chang HY Bohr VA Ried T Gozani O Chua KF (2008) SIRT6 is a histone H3 lysine 9 deacetylase that modulates telomeric chromatin. Nature 452: 492–496. 10.1038/nature06736 18337721 PMC2646112

[B174] Montefalcone G Tempesta S Rocchi M Archidiacono N (1999) Centromere repositioning. Genome Research 9: 1184–1188. 10.1101/gr.9.12.1184 10613840 PMC311001

[B175] Montero JJ López-Silanes I Megías D Fraga FM Castells-García Á Blasco MA (2018) TERRA recruitment of polycomb to telomeres is essential for histone trymethylation marks at telomeric heterochromatin. Nature Communications 9: 1548. 10.1038/s41467-018-03916-3 PMC5906467 29670078

[B176] Mosch K Franz H Soeroes S Singh PB Fischle W (2011) HP1 recruits activity-dependent neuroprotective protein to H3K9me3 marked pericentromeric heterochromatin for silencing of major satellite repeats. PLoS One 6(1): e15894. 10.1371/journal.pone.0015894 PMC3022755 21267468

[B177] Moyzis RK Buckingham JM Cram LS Dani M Deaven LL Jones MD Meyne J Ratliff RL Wu JR (1988) A highly conserved repetitive DNA sequence, (TTAGGG)n, present at the telomeres of human chromosomes. Proceedings of the National Academy of Sciences of the United States of America 85: 6622–6626. 10.1073/pnas.85.18.6622 3413114 PMC282029

[B178] Mozgová I Schrumpfova PP Hofr C Fajkus J (2008) Functional characterization of domains in AtTRB1, a putative telomere-binding protein in *Arabidopsis thaliana*. Phytochemistry 69: 1814–1819. 10.1016/j.phytochem.2008.04.001 18479720

[B179] Müller F Wicky C Spicher A Tobler H (1991) New telomere formation after developmentally regulated chromosomal breakage during the process of chromatin diminution in *Ascaris lumbricoides*. Cell 67(4): 815–822. 10.1016/0092-8674(91)90076-B 1934070

[B180] Nagaki K Murata M (2005) Characterization of cenH3 and centromere-associated DNA sequences in sugarcane. Chromosome Research 13: 195–203. 10.1007/s10577-005-0847-2 15861308

[B181] Nagaki K Talbert PB Zhong CX Dawe RK Henikoff S Jiang J (2003) Chromatin immunoprecipitation reveals that the 180-bp satellite repeat is the key functional DNA element of *Arabidopsis thaliana* centromeres. Genetics 163: 1221–1225. https://www.ncbi.nlm.nih.gov/pubmed/12663558 12663558 10.1093/genetics/163.3.1221 PMC1462492

[B182] Nagaki K Cheng Z Ouyang S Talbert PB Kim M Jones KM Henikoff S Buell CR Jiang J (2004) Sequencing of a rice centromere uncovers active genes. Nature Genetics 36(2): 138–145. 10.1038/ng1289 14716315

[B183] Nakano M Cardinale S Noskov VN Gassmann R Vagnarelli P Kandels-Lewis S Larionov V Earnshaw WC Masumoto H (2008) Inactivation of a human kinetochore by specific targeting of chromatin modifiers. Developmental Cell 14: 507–522. 10.1016/j.devcel.2008.02.001 18410728 PMC2311382

[B184] Nakayama J Rice JC Strahl BD Allis CD Grewal SI (2001) Role of histone H3 lysine 9 methylation in epigenetic control of heterochromatin assembly. Science 292: 110–113. 10.1126/science.1060118 11283354

[B185] Nasmyth K Haering CH (2009) Cohesin: its roles and mechanisms. Annual Review of Genetics 43: 525–558. 10.1146/annurev-genet-102108-134233 19886810

[B186] Nasuda S Hudakova S Schubert I Houben A Endo TR (2005) Stable barley chromosomes without centromeric repeats. Proceedings of the National Academy of Sciences of the United States of America 102: 9842–9847. 10.1073/pnas.0504235102 15998740 PMC1175009

[B187] Nergadze SG Farnung BO Wischnewski H Khoriauli L Vitelli V Chawla R Giulotto E Azzalin CM (2009) CpG-island promoters drive transcription of human telomeres. RNA 15: 2186–2194. 10.1261/rna.1748309 19850908 PMC2779677

[B188] Neumann P Yan H Jiang J (2007) The centromeric retrotransposons of rice are transcribed and differentially processed by RNA interference. Genetics 176: 749–761. 10.1534/genetics.107.071902 17409063 PMC1894605

[B189] Ng LJ Cropley JE Pickett HA Reddel RR Suter CM (2009) Telomerase activity is associated with an increase in DNA methylation at the proximal subtelomere and a reduction in telomeric transcription. Nucleic Acids Research 37: 1152–1159. 10.1093/nar/gkn1030 19129228 PMC2651807

[B190] Nislow C Ray E Pillus L (1997) SET1, a yeast member of the trithorax family, functions in transcriptional silencing and diverse cellular processes. Molecular Biology of the Cell 8: 2421–2436. 10.1091/mbc.8.12.2421 9398665 PMC25717

[B191] O’Sullivan RJ Kubicek S Schreiber SL Karlseder J (2010) Reduced histone histone biosynthesis and chromatin changes arising from a damage signal at telomeres. Nature Structural and Molecular Biology 17(10): 1218–25. 10.1038/nsmb.1897 PMC2951278 20890289

[B192] Ogrocká A Polanska P Majerova E Janeba Z Fajkus J Fojtova M (2014) Compromised telomere maintenance in hypomethylated *Arabidopsis thaliana* plants. Nucleic Acids Research 42: 2919–2931. 10.1093/nar/gkt1285 24334955 PMC3950684

[B193] Ohkuni K Kitagawa K (2011) Endogenous transcription at the centromere facilitates centromere activity in budding yeast. Current Biology 21: 1695–1703. 10.1016/j.cub.2011.08.056 22000103 PMC3218120

[B194] Oliveira LC Torres GA (2018) Plant centromeres: genetics, epigenetics and evolution. Molecular Biology Reports 45: 1491–1497. 10.1007/s11033-018-4284-7 30117088

[B195] Ostromyshenskii DI Chernyaeva EN Kuznetsova IS Podgornaya OI (2018) Mouse chromocenters DNA content: sequencing and *in silico* analysis. BMC Genomics 19(1): 151 10.1186/s12864-018-4534-z 29458329 PMC5819297

[B196] Peška V Schrumpfova PP Fajkus J (2011) Using the telobox to search for plant telomere binding proteins. Current Protein and Peptide Science 12: 75–83. 10.2174/138920311795684968 21348850

[B197] Peters AH O’Carroll D Scherthan H Mechtler K Sauer S Schofer C Weipoltshammer K Pagani M Lachner M Kohlmaier A Opravil S Doyle M Sibilia M Jenuwein T (2001) Loss of the Suv39h histone methyltransferases impairs mammalian heterochromatin and genome stability. Cell 107: 323–337. 10.1016/S0092-8674(01)00542-6 11701123

[B198] Piras FM Nergadze SG Magnani E Bertoni L Attolini C Khoriauli L Raimondi E Giulotto E (2010) Uncoupling of satellite DNA and centromeric function in the genus *Equus* PLoS Genetics 6:e1000845. 10.1371/journal.pgen.1000845 PMC2820525 20169180

[B199] Pisano S Marchioni E Galati A Mechelli R Savino M Cacchione S (2007) Telomeric nucleosomes are intrinsically mobile. Journal of Molecular Biology 369: 1153–1162. 10.1016/j.jmb.2007.04.027 17498745

[B200] Plohl M Meštrović N Mravinac B (2014) Centromere identity from the DNA point of view. Chromosoma 123: 313–325. 10.1007/s00412-014-0462-0 24763964 PMC4107277

[B201] Plohl M Luchetti A Meštrović N Mantovani B (2008) Satellite DNAs between selfishness and functionality: structure, genomics and evolution of tandem repeats in centromeric (hetero)chromatin. Gene 409: 72–82. 10.1016/j.gene.2007.11.013 18182173

[B202] Pluta AF Mackay AM Ainsztein AM Goldberg IG Earnshaw WC (1995) The centromere: hub of chromosomal activities. Science 270: 1591–1594. 10.1126/science.270.5242.1591 7502067

[B203] Podgornaya O Gavrilova E Stephanova V Demin S Komissarov A (2013) Large tandem repeats make up the chromosome bar code: a hypothesis. Advances in Protein Chemistry and Structural Biology 90: 1–30. 10.1016/B978-0-12-410523-2.00001-8 23582200

[B204] Porro A Feuerhahn S Reichenbach P Lingner J (2010) Molecular dissection of telomeric repeat-containing RNA biogenesis unveils the presence of distinct and multiple regulatory pathways. Molecular and Cellular Biology 30: 4808–4817. 10.1128/MCB.00460-10 20713443 PMC2950545

[B205] Porro A Feuerhahn S Delafontaine J Riethman H Rougemont J Lingner J (2014) Functional characterization of the TERRA transcriptome at damaged telomeres. Nature Communications 5: 5379. 10.1038/ncomms6379 PMC4264578 25359189

[B206] Postepska-Igielska A Krunic D Schmitt N Greulich-Bode KM Boukamp P Grummt I (2013) The chromatin remodelling complex NoRC safeguards genome stability by heterochromatin formation at telomeres and centromeres. EMBO reports 14(8): 704–710. 10.1038/embor.2013.87 23797874 PMC3736129

[B207] Probst AV Fransz PF Paszkowski J Scheid OM (2003) Two means of transcriptional reactivation within heterochromatin. Plant Journal 33: 743–749. 10.1046/j.1365-313X.2003.01667.x 12609046

[B208] Procházková-Schrumpfová P Fojtova M Fajkus J (2019) Telomeres in plants and humans: not so different, not so similar. Cells 8(1): E58. 10.3390/cells8010058 PMC6356271 30654521

[B209] Pucci F Gardano L Harrington L (2013) Short telomeres in ESCs lead to unstable differentiation. Cell Stem Cell 12: 479–486. 10.1016/j.stem.2013.01.018 23561444 PMC3629568

[B210] Quénet D Dalal Y (2014) A long non-coding RNA is required for targeting centromeric protein A to the human centromere. Elife 3: e03254. 10.7554/eLife.03254 PMC4145801 25117489

[B211] Quina AS Buschbeck M Di Croce L (2006) Chromatin structure and epigenetics. Biochemical Pharmacology 72(11): 1563–1569. 10.1016/j.bcp.2006.06.016 16836980

[B212] Ravi M Kwong PN Menorca RM Valencia JT Ramahi JS Stewart JL Tran RK Sundaresan V Comai L Chan SW (2010) The rapidly evolving centromere-specific histone has stringent functional requirements in *Arabidopsis thaliana*. Genetics 186(2): 461–471. 10.1534/genetics.110.120337 20628040 PMC2954480

[B213] Reinberg D Sims RJ III (2006) de FACTo nucleosome dynamics. J Biol Chem 281: 23297–23301. 10.1074/jbc.R600007200 16766522

[B214] Ribeiro T Marques A Novák P Schubert V Vanzela ALL Macas J Houben A Pedrosa-Harand A (2017) Centromeric and non-centromeric satellite DNA organization differs in holocentric *Rhynchospora* species. Chromosoma 126: 325–335. 10.1007/s00412-016-0616-3 27645892

[B215] Richards EJ Ausubel FM (1988) Isolation of a higher eukaryotic telomere from *Arabidopsis thaliana*. Cell 53: 127–136. 10.1016/0092-8674(88)90494-1 3349525

[B216] Riethman H Ambrosisni A Paul S (2005) Human subtelomere structure and variation. Chromosome Research 13: 505–515. 10.1007/s10577-005-0998-1 16132815

[B217] Riha K McKnight TD Fajkus J Vyskot B Shippen DE (2000) Analysis of the G-overhang structures on plant telomeres: Evidence for two distinct telomere architectures. Plant Journal 23: 633–641. 10.1046/j.1365-313x.2000.00831.x 10972889

[B218] Roberts A Pimentel H Trapnell C Pachter L (2011) Identification of novel transcripts in annotated genomes using RNA-Seq. Bioinformatics 27: 2325–2329. 10.1093/bioinformatics/btr355 21697122

[B219] Rodionov AV Lukina NA Galkina SA Solovei I Saccone S (2002) Crossing over in chicken oogenesis: cytological and chiasma-based genetic maps of the chicken lampbrush chromosome 1. Journal of Heredity 93: 125Y129. 10.1093/jhered/93.2.125 12140272

[B220] Rose NR Klose RJ (2014) Understanding the relationship between DNA methylation and histone lysine methylation. Biochimica et Biophysica Acta 1839: 1362–1372. 10.1016/j.bbagrm.2014.02.007 24560929 PMC4316174

[B221] Rosenfeld JA Wang Z Schones DE Zhao K Desalle R Zhang MQ (2009) Determination of enriched histone modifications in non-genic portions of the human genome. BMC Genomics 10: 143. 10.1186/1471-2164-10-143 PMC2667539 19335899

[B222] Rošić S Köhler F Erhardt S (2014) Repetitive centromeric satellite RNA is essential for kinetochore formation and cell division. Journal of Cell Biology 207(3): 335–349. 10.1083/jcb.201404097 25365994 PMC4226727

[B223] Rosin LF Mellone BG (2017) Centromeres drive a hard bargain. Trends Genetics 33: 101–117. 10.1016/j.tig.2016.12.001 PMC5467322 28069312

[B224] Roudier F Ahmed I Berard C Sarazin A Mary-Huard T Cortijo S Bouyer D Caillieux E Duvernois-Berthet E Al-Shikhley L Giraut L Després B Drevensek S Barneche F Dèrozier S Brunaud V Aubourg S Schnittger A Bowler C Martin-Magniette ML Robin S Caboche M Colot V (2011) Integrative epigenomic mapping defines four main chromatin states in *Arabidopsis*. EMBO Journal 30: 1928–1938. 10.1038/emboj.2011.103 21487388 PMC3098477

[B225] Roulland Y Ouararhni K Naidenov M Ramos L Shuaib M Syed SH Lone IN Boopathi R Fontaine E Papai G Tachiwana H Gautier T Skoufias D Padmanabhan K Bednar J Kurumizaka H Schultz P Angelov D Hamiche A Dimitrov S (2016) The Flexible Ends of CENP-A Nucleosome Are Required for Mitotic Fidelity. Molecular Cell 63: 674–685. 10.1016/j.molcel.2016.06.023 27499292

[B226] Sadeghi L Siggens L Svensson JP Ekwall K (2014) Centromeric histone H2B monoubiquitination promotes noncoding transcription and chromatin integrity. Nature Structural and Molecular Biology 21: 236–43. 10.1038/nsmb.2776 24531659

[B227] Saksouk N Barth TK Ziegler-Birling C Olova N Nowak A Rey E Mateos-Langerak J Urbach S Reik W Torres-Padilla ME Imhof A Déjardin J Simboeck E (2014) Redundant mechanisms to form silent chromatin at pericentromeric regions rely on BEND3 and DNA methylation. Molecular Cell 56(4): 580–594. 10.1016/j.molcel.2014.10.001 25457167

[B228] Saksouk N Simboeck E Déjardin J (2015) Constitutive heterochromatin formation and transcription in mammals. Epigenetics Chromatin 8: 3. 10.1186/1756-8935-8-3 PMC4363358 25788984

[B229] Sakuno T Tada K Watanabe Y (2009) Kinetochore geometry defined by cohesion within the centromere. Nature 458: 852–858. 10.1038/nature07876 19370027

[B230] Salmon ED Bloom K (2017) Tension sensors reveal how the kinetochore shares its load. BioEssays 39(7): 10.1002/bies.201600216 PMC5543933 28582586

[B231] Santenard A Ziegler-Birling C Koch M Tora L Bannister AJ Torres-Padilla ME (2010) Heterochromatin formation in the mouse embryo requires critical residues of the histone variant H3.3. Nature Cell Biology 12: 853–862. 10.1038/ncb2089 20676102 PMC3701880

[B232] Schalch T Steiner FA (2017) Structure of centromere chromatin: from nucleosome to chromosomal architecture. Chromosoma 126: 443–455. 10.1007/s00412-016-0620-7 27858158 PMC5509776

[B233] Schoeftner S Blasco MA (2008) Developmentally regulated transcription of mammalian telomeres by DNA-dependent RNA polymerase II. Nature Cell Biology 10: 228–236. 10.1038/ncb1685 18157120

[B234] Schrumpfova PP Kuchar M Palecek J Fajkus J (2008) Mapping of interaction domains of putative telomere-binding proteins AtTRB1 and AtPOT1b from *Arabidopsis thaliana*. FEBS Letters 582: 1400–1406. 10.1016/j.febslet.2008.03.034 18387366

[B235] Schueler MG Higgins AW Rudd MK Gustashaw K Willard HF (2001) Genomic and genetic definition of a functional human centromere. Science 294: 109–115. 10.1126/science.1065042 11588252

[B236] Sen D Gilbert W (1988) Formation of parallel four-stranded complexes by guanine-rich motifs in DNA and its implications for meiosis. Nature 334: 364–366. 10.1038/334364a0 3393228

[B237] Shakirov EV Surovtseva YV Osbun N Shippen DE (2005) The *Arabidopsis* Pot1 and Pot2 proteins function in telomere length homeostasis and chromosome end protection. Molecular and Cellular Biology 257725–257733. 10.1128/MCB.25.17.7725-7733.2005 PMC1190295 16107718

[B238] Shang WH Hori T Westhorpe FG Godek KM Toyoda A Misu S Monma N Ikeo K Carroll CW Takami Y Fujiyama A Kimura H Straight AF Fukagawa T (2016) Acetylation of histone H4 lysine 5 and 12 is required for CENP-A deposition into centromeres. Nature Communications 7: 13465. 10.1038/ncomms13465 PMC5097169 27811920

[B239] Shang WH Hori T Martins NM Toyoda A Misu S Monma N Hiratani I Maeshima K Ikeo K Fujiyama A Kimura H Earnshaw WC Fukagawa T (2013) Chromosome engineering allows the efficient isolation of vertebrate neocentromeres. Developmental Cell 24: 635–648. 10.1016/j.devcel.2013.02.009 23499358 PMC3925796

[B240] Shanower GA Muller M Blanton JL Honti V Gyurkovics H Schedl P (2005) Characterization of the grappa gene, the Drosophila histone H3 lysine 79 methyltransferase. Genetics 169: 173–184. 10.1534/genetics.104.033191 15371351 PMC1448877

[B241] She C-W Wei L Jiang X-H (2017) Molecular cytogenetic characterization and comparison of the two cultivated *Canavalia* species (Fabaceae). Comparative Cytogenetics 11(4): 579–600. 10.3897/compcytogen.v11i4.13604 29114355 PMC5672272

[B242] Shema-Yaacoby E Nikolov M Haj-Yahya M Siman P Allemand E Yamaguchi Y Muchardt C Urlaub H Brik A Oren M Fischle W (2013) Systematic identification of proteins binding to chromatin-embedded ubiquitylated H2B reveals recruitment of SWI/SNF to regulate transcription. Cell Reports 4: 601–608. 10.1016/j.celrep.2013.07.014 23933260

[B243] Shi D-Q Ali I Tang J Yang W-C (2017) New insights into 5hmC DNA modification: generation, distribution and function. Frontiers in Genetics 8: 100. 10.3389/fgene.2017.00100 PMC5515870 28769976

[B244] Shuaib M Ouararhni K Dimitrov S Hamiche A (2010) HJURP binds CENP-A via a highly conserved N-terminal domain and mediates its deposition at centromeres. Proceedings of the National Academy of Sciences of the United States of America 107: 1349–1354. 10.1073/pnas.0913709107 20080577 PMC2824361

[B245] Simon L Voisin M Tatout C Probst AV (2015) Structure and function of centromeric and pericentromeric heterochromatin in *Arabidopsis thaliana* Front Plant Science 6: 1049. 10.3389/fpls.2015.01049 PMC4663263 26648952

[B246] Simonet T Zaragosi LE Philippe C Lebrigand K Schouteden C Augereau A Bauwens S Ye J Santagostino M Giulotto E Magdinier F Horard B Barbry P Waldmann R Gilson E (2011) The human TTAGGG repeat factors 1 and 2 bind to a subset of interstitial telomeric sequences and satellite repeats. Cell Research 21: 1028–1038. 10.1038/cr.2011.40 21423270 PMC3193489

[B247] Smogorzewska A de Lange T (2004) Regulation of telomerase by telomeric proteins. Annual Review of Biochemistry 73: 177–208. 10.1146/annurev.biochem.73.071403.160049 15189140

[B248] Smurova K Wulf PD (2018) Centromere and pericentromere transcription: roles and regulation … in sickness and in health. Frontiers in Genetics 9: 674. 10.3389/fgene.2018.00674 PMC6309819 30627137

[B249] Sollier J Cimprich KA (2015) Breaking bad: R-loops and genome integrity. Trend in Cell Biology 25: 514–522. 10.1016/j.tcb.2015.05.003 PMC4554970 26045257

[B250] Solovei I Thanisch K Feodorova Y (2016) How to rule the nucleus: *divide et impera*. Current Opinion in Cell Biology 40: 47–59. 10.1016/j.ceb.2016.02.014 26938331

[B251] Song QX Lu X Li QT Chen H Hu XY Ma B Zhang WK Chen SY Zhang JS (2013) Genome-wide analysis of DNA methylation in soybean. Molecular Plant 6(6): 1961–1974. 10.1093/mp/sst123 23966636

[B252] Srivastava S Foltz DR (2018) Posttranslational modifications of CENP-A: marks of distinction. Chromosoma 127(3): 279–290. 10.1007/s00412-018-0665-x 29569072 PMC6082721

[B253] Steiner FA Henikoff S (2015) Diversity in the organization of centromeric chromatin. Current Opinion in Genetics and Development 31: 28–35. 10.1016/j.gde.2015.03.010 25956076

[B254] Stroud H Greenberg MV Feng S Bernatavichute YV Jacobsen SE (2013) Comprehensive analysis of silencing mutants reveals complex regulation of the *Arabidopsis* methylome. Cell 152: 352–364. 10.1016/j.cell.2012.10.054 23313553 PMC3597350

[B255] Subirana JA Albà MM Messeguer X (2015) High evolutionary turnover of satellite families in *Caenorhabditis* BMC Evolutionary Biology 15: 218. 10.1186/s12862-015-0495-x PMC4595182 26438045

[B256] Sullivan BA (2002) Centromere round-up at the heterochromatin corral. Trends Biotechnology 20(3): 89–92. 10.1016/S0167-7799(02)01902-9 11841851

[B257] Sullivan BA Karpen GH (2004) Centromeric chromatin exhibits a histone modification pattern that is distinct from both euchromatin and heterochromatin. Nature Structural & Molecular Biology 11: 1076–1083. 10.1038/nsmb845 PMC1283111 15475964

[B258] Sullivan BA Schwartz S (1995) Identification of centromeric antigens in dicentric Robertsonian translocations: CENP-C and CENP-E are necessary components of functional centromeres. Human Molecular Genetics 4: 2189–2197. 10.1093/hmg/4.12.2189 8634687

[B259] Tachiwana H Kagawa W Shiga T Osakabe A Miya Y Saito K Hayashi-Takanaka Y Oda T Sato M Park SY Kimura H Kurumizaka H (2011) Crystal structure of the human centromeric nucleosome containing CENP-A. Nature 476: 232–235. 10.1038/nature10258 21743476

[B260] Tek AL Kashihara K Murata M Nagaki K (2010) Functional centromeres in soybean include two distinct tandem repeats and a retrotransposon. Chromosome Research 18: 337–347. 10.1007/s10577-010-9119-x 20204495

[B261] Topp CN Zhong CX Dawe RK (2004) Centromere-encoded RNAs are integral components of the maize kinetochore. Proceedings of the National Academy of Sciences of the United States of America 101: 15986–15991. 10.1073/pnas.0407154101 15514020 PMC528775

[B262] Topp CN Okagaki RJ Melo JR Kynast RG Phillips RL Dawe RK (2009) Identification of a maize neocentromere in an oat-maize addition line. Cytogenetics and Genome Research 124: 228–238. 10.1159/000218128 19556776 PMC2813801

[B263] Torres GA Gong Z Iovene M Hirsch CD Buell CR Bryan GJ Novák P Macas J Jiang J (2011) Organization and evolution of subtelomeric satellite repeats in the potato genome. G3 (Bethesda) 1(2): 85–92. 10.1534/g3.111.000125 PMC3276127 22384321

[B264] Tran DT Cao HX Jovtchev G Neumann P Novak P Fojtova M Vu GTH Macas J Fajkus J Schubert I Fuchs J (2015) . Centromere and telomere sequence alterations reflect the rapid genome evolution within the carnivorous plant genus *Genlisea* Plant Journal 84 1087–1099. 10.1111/tpj.13058 26485466

[B265] Vaquero-Sedas MI Vega-Palas MA (2011) On the chromatin structure of eukaryotic telomeres. Epigenetics 6: 1055–1058. 10.4161/epi.6.9.16845 21822057 PMC3225743

[B266] Vaquero-Sedas MI Gámez-Arjona FM Vega-Palas MA (2011) *Arabidopsis thaliana* telomeres exhibit euchromatic features. Nucleic Acids Research 39(6): 2007–17. 10.1093/nar/gkq1119 21071395 PMC3064777

[B267] Vaquero-Sedas MI Luo CY Vega-Palas MA (2012) Analysis of the epigenetic status of telomeres by using ChIP-seq data. Nucleic Acids Research 40: e163. 10.1093/nar/gks730 PMC3505975 22855559

[B268] Vega-Vaquero A Bonora G Morselli M Vaquero-Sedas MI Rubbi L Pellegrini M Vega-Palas MA (2016) Novel features of telomere biology revealed by the absence of telomeric DNA methylation. Genome Research 26: 1047–1056. 10.1101/gr.202465.115 27405804 PMC4971770

[B269] Ventura M Antonacci F Cardone MF Stanyon R D’Addabbo P Cellamare A Sprague LJ Eichler EE Archidiacono N Rocchi M (2007) Evolutionary formation of new centromeres in macaque. Science 316: 243–246. 10.1126/science.1140615 17431171

[B270] Verdaasdonk JS Bloom K (2011) Centromeres: unique chromatin structures that drive chromosome segregation. Nature Reviews Molecular Cell Biology 12(5): 320–332. 10.1038/nrm3107 21508988 PMC3288958

[B271] Vermaak D Malik HS (2009) Multiple roles for heterochromatin protein 1 genes in *Drosophila*. Annual Review of Genetics 43: 467–492. 10.1146/annurev-genet-102108-134802 19919324

[B272] Vrbsky J Akimcheva S Watson JM Turner TL Daxinger L Vyskot B Aufsatz W Riha K (2010) siRNA-mediated methylation of *Arabidopsis* telomeres. PLoS Genetics 6: e1000986. 10.1371/journal.pgen.1000986 PMC2883606 20548962

[B273] Wang F Podell ER Zaug AJ Yang Y Baciu P Cech TR Lei M (2007) The POT1-TPP1 telomere complex is a telomerase processivity factor. Nature 445(7127): 506–510. 10.1038/nature05454 17237768

[B274] Wang F Ulyanova NP van der Waal MS Patnaik D Lens SMA Higgins JMG (2011) A positive feedback loop involving haspin and aurora B promotes CPC accumulation at centromeres in mitosis. Current Biology 21: 1061–1069. 10.1016/j.cub.2011.05.016 21658950 PMC3118923

[B275] Wang G Li H Cheng Z Jin W (2013) A novel translocation event leads to a recombinant stable chromosome with interrupted centromeric domains in rice. Chromosoma 122: 295–303. 10.1007/s00412-013-0413-1 23625520

[B276] Wang SS Zakian VA (1990) Telomere-telomere recombination provides an express pathway for telomere acquisition. Nature 345: 456–458. 10.1038/345456a0 2111466

[B277] Wang Z Zang C Rosenfeld JA Schones DE Barski A Cuddapah S Cui K Roh TY Peng W Zhang MQ Zhao K (2008) Combinatorial patterns of histone acetylations and methylations in the human genome. Nature Genetics 40: 897–903. 10.1038/ng.154 18552846 PMC2769248

[B278] Warburton PE Haaf T Gosden J Lawson D Willard HF (1996) Characterization of a Chromosome-Specific Chimpanzee Alpha Satellite Subset: Evolutionary Relationship to Subsets on Human Chromosomes. Genomics 33: 220–228. 10.1006/geno.1996.0187 8660971

[B279] Weber SA Gerton JL Polancic JE DeRisi JL Koshland D Megee PC (2004) The Kinetochore is an enhancer of pericentric cohesin binding. PLoS Biology 2(9): e260. 10.1371/journal.pbio.0020260 PMC490027 15309047

[B280] White MJD (1973) Animal Cytology and Evolution. Cambridge University Press; Cambridge.

[B281] Willard HF (1990) Trends centromeres of mammalian chromosomes. Genetics 6(12): 410–416. 10.1016/0168-9525(90)90302-M 2087784

[B282] Williams BC Murphy TD Goldberg ML Karpen GH (1998) Neocentromere activity of structurally acentric mini-chromosomes in *Drosophila*. Nature Genetics 18: 30–37. 10.1038/ng0198-30 9425896

[B283] Wrench DI Kethley JB Norton RA (1994) Cytogenetics of holokinetic chromosomes and inverted meiosis: keys to the evolutionary success of mites, with generalization on eukaryotes. In: Houck MA (Ed.) Mites: Ecological and evolutionary analysye of life history patterns.Chapman & Hall, New York, 282–343. 10.1007/978-1-4615-2389-5_11

[B284] Wu YF Kikuchi S Yan HH Zhang WL Rosenbaum H Iniguez AL Jiang JM (2011) Euchromatic subdomains in rice centromeres are associated with genes and transcription. Plant Cell 23: 4054–4064. 10.1105/tpc.111.090043 22080597 PMC3246336

[B285] Xie X Shippen DE (2018) DDM1 guards against telomere truncation in *Arabidopsis*. Plant Cell Reports 37(3): 501–513. 10.1007/s00299-017-2245-6 29392401 PMC5880217

[B286] Xu YM Du JY Lau AT (2014) Posttranslational modifications of human histone H3: an update. Proteomics 14: 2047–2060. 10.1002/pmic.201300435 25044606

[B287] Yamagata K Yamazaki T Miki H Ogonuki N Inoue K Ogura A Baba T (2007) Centromeric DNA hypomethylation as an epigenetic signature discriminates between germ and somatic cell lineages. Developmental Biology 312: 419–426. 10.1016/j.ydbio.2007.09.041 17964565

[B288] Yamagishi Y Honda T Tanno Y Watanabe Y (2010) Two histone marks establish the inner centromere and chromosome bi-orientation. Science 330: 239–243. 10.1126/science.1194498 20929775

[B289] Yan H Kikuchi S Neumann P Zhang W Wu Y Chen F Jiang J (2010) Genome-wide mapping of cytosine methylation revealed dynamic DNA methylation patterns associated with genes and centromeres in rice. Plant Journal 63: 353–365. 10.1111/j.1365-313X.2010.04246.x 20487381

[B290] Yan HH Jin WW Nagaki K Tian S Ouyang S Buell CR Talbert PB Henikoff S Jiang JM (2005) Transcription and histone modifications in the recombination-free region spanning a rice centromere. Plant Cell 17: 3227–3238. 10.1105/tpc.105.037945 16272428 PMC1315366

[B291] Yehezkel S Segev Y Viegas-Pequignot E Skorecki K Selig S (2008) Hypomethylation of subtelomeric regions in ICF syndrome is associated with abnormally short telomeres and enhanced transcription from telomeric regions. Human Molecular Genetics 17: 2776–2789. 10.1093/hmg/ddn177 18558631

[B292] Yelagandula R Stroud H Holec S Zhou K Feng S Zhong X Muthurajan UM Nie X Kawashima T Groth M Luger K Jacobsen SE Berger F (2014) The histone variant H2A.W defines heterochromatin and promotes chromatin condensation in *Arabidopsis*. Cell 158(1): 98–109. 10.1016/j.cell.2014.06.006 24995981 PMC4671829

[B293] Yi Q Chen Q Liang C Yan H Zhang Z Xiang X Zhang M Qi F Zhou L Wang F (2018) HP1 links centromeric heterochromatin to centromere cohesion in mammals. EMBO Reports 19: e45484. 10.15252/embr.201745484 PMC5891435 29491004

[B294] Zahler AM Williamson JR Cech TR Prescott DM (1991) Inhibition of telomerase by G-quartet DNA structures. Nature 350(6320): 718–20. 10.1038/350718a0 2023635

[B295] Zakrzewski F Schmidt T Weber B (2013) A molecular cytogenetic analysis of the structure, evolution, and epigenetic modifications of major DNA sequences in centromeres of *Beta species*. In: Jiang J Birchler JA (eds) Plant centromere biology.UK Wiley-Blackwell, Chichester, 39–55. 10.1002/9781118525715.ch4

[B296] Zemach A McDaniel IE Silva P Zilberman D (2010) Genome-wide evolutionary analysis of eukaryotic DNA methylation. Science 328(5980): 916–919. 10.1126/science.1186366 20395474

[B297] Zhang H Lang Z Zhu JK (2018) Dynamics and function of DNA methylation in plants. Nature Reviews Molecular Cell Biology 19(8): 489–506. 10.1038/s41580-018-0016-z 29784956

[B298] Zhang W Lee HR Koo DH Jiang J (2008) Epigenetic modification of centromeric chromatin: hypomethylation of DNA sequences in the cenH3-associated chromatin in *Arabidopsis thaliana* and maize. Plant Cell 20: 25–34. 10.1105/tpc.107.057083 18239133 PMC2254920

[B299] Zhang WL Friebe B Gill BS Jiang JM (2010) Centromere inactivation and epigenetic modifications of a plant chromosome with three functional centromeres. Chromosoma 119: 553–563. 10.1007/s00412-010-0278-5 20499078

[B300] Zhang W Yeung CHL Wu L Yuen KWY (2017) E3 ubiquitin ligase Bre1 couples sister chromatid cohesion establishment to DNA replication in *Saccharomyces cerevisiae* ELife 6: e28231. 10.7554/eLife.28231.020 PMC5699866 29058668

[B301] Zhang H Koblizkova A Wang K Gong Z Oliveira L Torres GA Wu Y Zhang W Novák P Buell CR Macas J (2014) Boom-bust turnovers of megabase-sized centromeric DNA in *Solanum* species: rapid evolution of DNA sequences associated with centromeres. Plant Cell 26: 1436–1447. 10.1105/tpc.114.123877 24728646 PMC4036563

[B302] Zhimulev IF Zykova TY Goncharov FP Khoroshko VA Demakova OV Semeshin VF Pokholkova GV Boldyreva LV Demidova DS Babenko VN Demakov SA Belyaeva ES (2014) Genetic organization of interphase chromosome bands and interbands in *Drosophila melanogaster* PLoS One 9(7): e101631. 10.1371/journal.pone.0101631 PMC4114487 25072930

[B303] Zhong CX Marshall JB Topp C Mroczek R Kato A Nagaki K Birchler JA Jiang J Dawe RK (2002) Centromeric retroelements and satellites interact with maize kinetochore protein cenH3. Plant Cell 14: 2825–2836. 10.1105/tpc.006106 12417704 PMC152730

[B304] Zhong Z Shiue L Kaplan S de Lange T (1992) A mammalian factor that binds telomeric TTAGGG repeats *in vitro*. Molecular and Cellular Biology 12: 4834–4843. 10.1128/MCB.12.11.4834 1406665 PMC360416

[B305] Zhu H Duan CG Hou WN Du QS Lv DQ Fang RX Guo HS (2011) Satellite RNA-derived small interfering RNA satsiR-12 targeting the 39 untranslated region of Cucumber mosaic virus triggers viral RNAs for degradation. Journal of Virology 85: 13384–13397. 10.1128/JVI.05806-11 21994448 PMC3233178

[B306] Zilberman D Coleman-Derr D Ballinger T Henikoff S (2008) Histone H2A.Z and DNA methylation are mutually antagonistic chromatin marks. Nature 456(7218): 125–129. 10.1038/nature07324 18815594 PMC2877514

[B307] Zlotina A Galkina S Krasikova A Crooijmans RP Groenen MAM Gaginskaya ER Deryusheva S (2012) Centromer positions in chicken and Japanese quail chromosomes: de novo centromere formation versus pericentric inversions. Chromosome Research 20(8): 1017–1032. 10.1007/s10577-012-9319-7 23143647

